# Radiomics and *MGMT* promoter methylation for prognostication of newly diagnosed glioblastoma

**DOI:** 10.1038/s41598-019-50849-y

**Published:** 2019-10-08

**Authors:** Takahiro Sasaki, Manabu Kinoshita, Koji Fujita, Junya Fukai, Nobuhide Hayashi, Yuji Uematsu, Yoshiko Okita, Masahiro Nonaka, Shusuke Moriuchi, Takehiro Uda, Naohiro Tsuyuguchi, Hideyuki Arita, Kanji Mori, Kenichi Ishibashi, Koji Takano, Namiko Nishida, Tomoko Shofuda, Ema Yoshioka, Daisuke Kanematsu, Yoshinori Kodama, Masayuki Mano, Naoyuki Nakao, Yonehiro Kanemura

**Affiliations:** 1 Department of Neurosurgery, Wakayama Rosai Hospital, Wakayama, 640-8505 Japan; 2 Kansai Molecular Diagnosis Network for CNS Tumors, Osaka, 540-0006 Japan; 3 Department of Neurological Surgery, Wakayama Medical University School of Medicine, Wakayama, 641-0012 Japan; 4 Department of Neurosurgery, Osaka International Cancer Institute, Osaka, 541-8567 Japan; 5 Department of Neurosurgery, Osaka University Graduate School of Medicine, Suita, 565-0871 Japan; 6 Department of Neurosurgery, National Hospital Organization Osaka National Hospital, Osaka, 540-0006 Japan; 7 Department of Neurosurgery, Kansai Medical University, Hirakata, 573-1191 Japan; 8 Moriuchi Clinic of Neurosurgery, Izumiotsu, Osaka 595-0024 Japan; 9 Department of Neurosurgery, Osaka City University Graduate School of Medicine, Osaka, 545-0051 Japan; 10 Department of Neurosurgery, Kindai University Faculty of Medicine, Sayama, 589-8511 Japan; 11 Department of Neurosurgery, Kansai Rosai Hospital, Amagasaki, 660-8511 Japan; 12 Department of Neurosurgery, Osaka City General Hospital, Osaka, 534-0021 Japan; 13 Department of Neurosurgery, Toyonaka Municipal Hospital, Toyonaka, 560-8565 Japan; 14 Department of Neurosurgery, Tazuke Kofukai Foundation, Medical Research Institute, Kitano Hospital, Osaka, 530-8480 Japan; 15 Division of Stem Cell Research, Department of Biomedical Research and Innovation, Institute for Clinical Research, National Hospital Organization Osaka National Hospital, Osaka, 540-0006 Japan; 16 Division of Regenerative Medicine Department of Biomedical Research and Innovation, Institute for Clinical Research, National Hospital Organization Osaka National Hospital, Osaka, 540-0006 Japan; 17 Division of Pathology Network, Kobe University Graduate School of Medicine, Kobe, 650-0017 Japan; 18 Department of Central Laboratory and Surgical Pathology, National Hospital Organization Osaka National Hospital, Osaka, 540-0006 Japan; 19 Department of Biomedical Research and Innovation, Institute for Clinical Research, National Hospital Organization Osaka National Hospital, Osaka, 540-0006 Japan

**Keywords:** Prognostic markers, Cancer imaging

## Abstract

We attempted to establish a magnetic resonance imaging (MRI)-based radiomic model for stratifying prognostic subgroups of newly diagnosed glioblastoma (GBM) patients and predicting O (6)-methylguanine-DNA methyltransferase promotor methylation (*pMGMT*-met) status of the tumor. Preoperative MRI scans from 201 newly diagnosed GBM patients were included in this study. A total of 489 texture features including the first-order feature, second-order features from 162 datasets, and location data from 182 datasets were collected. Supervised principal component analysis was used for prognostication and predictive modeling for *pMGMT*-met status was performed based on least absolute shrinkage and selection operator regression. 22 radiomic features that were correlated with prognosis were used to successfully stratify patients into high-risk and low-risk groups (*p* = 0.004, Log-rank test). The radiomic high- and low-risk stratification and *pMGMT* status were independent prognostic factors. As a matter of fact, predictive accuracy of the *pMGMT* methylation status was 67% when modeled by two significant radiomic features. A significant survival difference was observed among the combined high-risk group, combined intermediate-risk group (this group consists of radiomic low risk and *pMGMT*-unmet or radiomic high risk and *pMGMT*-met), and combined low-risk group (*p* = 0.0003, Log-rank test). Radiomics can be used to build a prognostic score for stratifying high- and low-risk GBM, which was an independent prognostic factor from *pMGMT* methylation status. On the other hand, predictive accuracy of the *pMGMT* methylation status by radiomic analysis was insufficient for practical use.

## Introduction

Glioblastoma (GBM) shows poor prognosis despite development of multimodal treatment including surgery, radiation therapy, and chemotherapy. O (6)-methylguanine-DNA methyltransferase (*MGMT*) promotor methylation (*pMGMT*-met) is a favorable prognostic factor in GBM patients, and patients with GBM and *pMGMT*-met benefit from temozolomide^1^. On the other hand, meta-analysis of previous clinical trials revealed that clinical features such as age, neurological status, and extent of tumor removal or residual tumor volume post-surgery are independent prognostic factors in GBM. These facts indicate that prognosis of this malignant disease is impacted by both the biological nature of the disease and the clinical manifestation of the patients. Accurate prognostication for each patient is necessary not only to determine the most appropriate individual treatment strategy but also to identify prognostic factors for patient stratification in clinical trials. Furthermore, in clinical practice, identifying patients who will potentially have a good outcome despite harboring this devastating disease is of great benefit for not only the patients and their families but also for primary physicians to provide hope while the patient goes through the demanding treatment procedures.

In this investigation, we retrieved as much information as possible from the initial magnetic resonance images (MRIs) for patients with GBM by use of radiomics^2–4^. The hypothesis that radiomics of GBM could build a radiologically derived prognostic score was tested with respect to the tumor’s *pMGMT*-met status.

## Materials and Methods

### Patient cohort and inclusion criteria

This study was performed in accordance with the principles of the Helsinki Declaration and was approved by the internal ethical review boards of Wakayama Medical University, Osaka International Cancer Institute, and all collaborative institutes, the list of which can be found in the acknowledge section. Written informed consent was obtained from all patients.

Inclusion criteria for the present study were as follows: new in-house diagnosis of GBM with fresh or frozen tissue available for genomic analysis, and preoperative MRI available including T1-weighted images (WI), T2WI, and gadolinium-enhanced (Gd) T1WI. Finally, 201 cases from 10 institutions that belong to the Kansai Molecular Diagnosis Network for central nervous system tumors were eligible for analysis. Frozen or fresh tumor samples were obtained at the time of surgery, and tumor genomic DNA was extracted from those tissues for genetic analysis.

### Genetic analysis and integrated diagnosis

**Figure 1:**
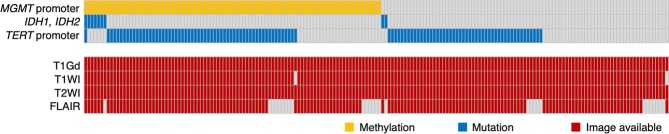
Overview of the analyzed cohort with landscape of genetic information. Genetic status is shown by color as indicated.

Genetic analyses were performed in the Osaka National Hospital according to the procedures previously described^5^. Briefly, the methylation status of the *MGMT* promoter was analyzed by quantitative methylation-specific PCR after bisulfite modification of genomic DNA, and we used a cut-off of ≥1% for *MGMT* promoter methylation^5^. The presence of hotspot mutations in *IDH1* (R132) and *IDH2* (R172)^6^ and the two mutation hotspots in the *TERT* promoter^7^ were analyzed by Sanger sequencing. The copy number status of 1p-19q was determined by multiplex ligation-dependent probe amplification (Oligodendroglioma 1p-19q probemix and EK1 reagent kit, MRC-Holland, Amsterdam, the Netherlands). Central pathology review was performed by a senior board-certified neuropathologist (Y.K.), and integrated diagnosis and WHO grading were made based on the 2016 WHO Classification of Tumors of the CNS (2016 WHO). Patient characteristics are listed in Fig. 1 and Supplementary Dataset.

### MRI data and clinical information

All MRI analyzed in the present study were preoperatively acquired using either 1.5- or 3.0-T MRI scanners according to the protocols in each institution. Magnetic field strength of the scanners used is detailed in Supplementary Dataset. Gd-T1WIs were available in 181 cases, T1WIs in 179 cases, and T2WIs in 181 cases. Overall survival was determined as the time from the date of the initial surgery for diagnosis to the date of death or the latest follow-up. Progression free survival was determined as the time from the date of the initial surgery for diagnosis to the date when first progression was identified or the latest follow-up. Visual inspection of each images was conducted in advance to sending these images for radiomics analysis in order to ensure that collected images are qualified for being used as GBM diagnosis. Clinical information such as age, sex, KPS at initial presentation and type of surgery performed was collected as well as details of post-surgical treatment including use of chemotherapy and/or radiation therapy. As for surgery, gross total removal was considered when more than 90% of the bulk of the tumor was surgically removed whereas partial removal was considered as significant amount of tumor being removed but not reaching more than 90% of it. Biopsy was defied as surgery aiming only histological confirmation of the tumor with no intention for cytoreduction of the tumor. Details can be found in Supplementary Dataset.

### Radiomics

Radiomic analyses (radiomics) were conducted using image analyzing software developed in-house in combination with the Oxford Centre for Functional MRI of the Brain (FMRIB) Linear Image Registration Tool (FLIRT) provided by FMRIB Software Library (FSL)^8–10^. The in-house software was developed in Matlab (Mathworks, Natick, MA), and seamless data transfer was carried out between Matlab-based in-house software and FSL via FSL integration into Matlab. All Digital Imaging and Communications in Medicine format images were first converted to the Neuroimaging Informatics Technology Initiative format using MRIConvert (University of Oregon Lewis Center for Neuroimaging: http://lcni.uoregon.edu/~jolinda/MRIConvert/), followed by 256 gray-scale level conversion. Non-contrast T1WI and Gd-T1WI voxels that were in the top 0.1% in intensity were deleted as they were mainly high signal noise, and the remaining 99.9% were reallocated in 256 gray scale. For T2WI, 100% of the data range was reallocated in 256 gray scale. This procedure was necessary for intensity normalization across all images acquired by different MRI scanners. Previous study by the authors conducting radiomics in WHO grade 2 and 3 gliomas revealed that the above-mentioned intensity normalization was most suitable for further analysis^11^. Furthermore, T2Edge images were constructed by applying a Prewitt filter to T2WI. Gdzscore images were also constructed by performing a voxel-wise contrast enhancement calculation using non-contrast and Gd-T1WI. Tumors were delineated by manually tracing contrast enhancing lesions on Gd-T1WI and high-intensity lesions on T2WI in three dimensions by experienced surgical neuro-oncologists (T.S. and M.K.) to create two different voxels of interest (VOIs). A small subset of data was used for assessing interobserver agreement. VOIs of both Gd-T1WI and T2WI for case number 1, 2, 5, 6, 8, 9 were created by both of the neuro-oncologists and Dice index was calculated. Dice index for Gd-T1WI-VOI was 0.88 ± 0.05 and that for T2WI was 0.86 ± 0.03, indicating acceptable interobserver agreement (both in mean ± standard deviation).

**Figure 2:**
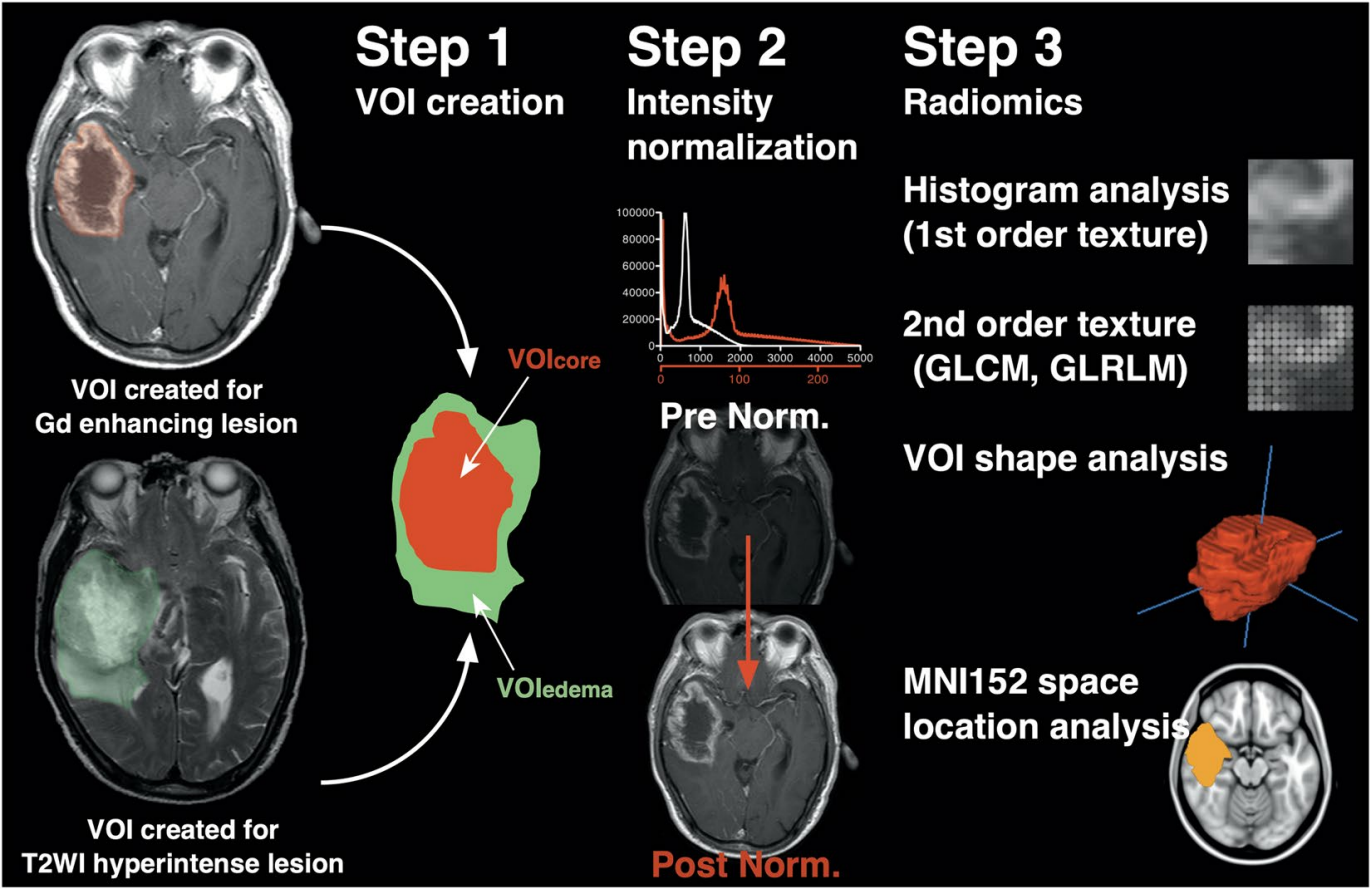
Illustration showing the workflow for image analysis. Two types of VOIs were created based on Gd enhancement of the tumor and edema lesion identification on T2-weighted images. Both VOIs were co-registered, and VOI_core_ and VOI_edema_ were generated. Subsequently, intensity normalization of all images was performed, and first-order and second-order texture analysis, VOI shape analysis, and location analysis were performed.

After VOIs were created, all different image sequences obtained from a single subject were co-registered to each other using a mutual information algorithm with 12 degrees of freedom transformation with FSL-FLIRT to obtain transformation matrices of different image sequences. Three-dimensional lesion VOIs modeled on Gd-T1WI and T2WI as mentioned above were deformed and resliced using the obtained transformation matrices via FSL-FLIRT for each specific image sequence. VOIs created on T2WI were subtracted from VOIs on Gd-T1WI to obtain VOIs for edema. VOIs created on Gd-T1WI were further denoted as “VOI_core_,” and the VOIs on T2WI subtracted from VOIs on Gd-T1WI as “VOI_edema_”. Three different aspects of texture features of the two VOIs were measured on T1WI, T2WI, Gd-T1WI, T2Edge, and Gdzscore image series, i.e., histogram-based first-order texture, second-order texture, and shape characteristics of the VOIs (Fig. 2).

Furthermore, T2WIs and Gd-T1WIs were registered to a 1.0-mm isotropic, high-resolution T1-weighted brain atlas provided by MNI152 using a mutual information algorithm with 12 degrees of freedom transformation with FSL-FLIRT. VOI_core_ and VOI_edema_ were then registered onto MNI152 by using the obtained transformation matrices. This procedure was necessary to perform lesion mapping of the VOIs on the standard MNI152 space. It should be noted that tissue classification i.e., delineation of VOI_core_ and VOI_edema_ was performed prior to spatial normalization and spatial normalization of these VOIs were performed using transformation matrices calculated between the whole brain of the patient and MNI152. Thus, eliminating any contamination of spatial normalization procedure into the process of tissue classification except for the fact that non-linear correction of the distorted brain by the lesion was not performed. The workflow for radiomics is illustrated in Fig. 2 and the calculated radiomic values are listed in Supplementary Table.

### Statistical analysis and predictive modeling

Statistical analysis was performed by M.K. using JMP Pro ver. 13 (SAS, Cary, NC). Survival analysis was performed with Wald test for continuous variables such as age and KPS and the Kaplan-Meier method for nominal variables. Multiple group comparison was performed by proportional hazard.

Supervised principal component analysis (SPCA) was used for prognostication using the referenced method^2^. SPCA was originally developed to identify subsets of patients with different survival outcome from a large set of available data such as gene expression profiles^12^ and has been successfully used for this purpose^13–15^. In this context, large data set such as gene expression profiles can be considered equivalent to radiomic data and although SPCA has some limitations such as limited number of subclass identification and ignoring potentially significant features^12^, SPCA has been shown to effectively perform in predicting overall survival in glioma radiomic study^2^. This analysis was performed on R using the Superpc package (https://cran.r-project.org/web/packages/superpc/superpc.pdf). The threshold for constructing a survival prediction was searched by 10-fold outer-loop-cross-validation using Superpc and a threshold parameter of 1.69 was achieved as the best tuned parameter for the Supervised Principal Component Predictor model. Importance score of each radiomic feature was calculated with the threshold hold of Supervised Principal Component Predictor model set as 1.69, enabling visualization of significant radiomic features predictive of patient survival. Finally, a binary radiomic risk classification was achieved using the default and parameters^12^ as suggested in the Superpc reference manual of the superpc.predict function. More specifically, n.components of 1 and prediction.type of discrete were used with the threshold set to 1.69 as mentioned above. The Superpc will in the end produce an “object”, which could be considered as a complex sum of algorithm that enables to classify each individual patient into either high- or low-radiomic risk groups according to the arguments which, in the current study, are the radiomic features. The most important concept that should be reminded is that this approach is designed to stratify high-risk (which also means short living) patients by comparing the survival dataset and the radiomics features.

Predictive modeling for *pMGMT*-met and long-term survivors, which is a different analysis from prognostication mentioned-above was performed based on the least absolute shrinkage and selection operator (LASSO) method to select features that were most significant to build predictive models for identifying patients that live longer than a given duration. λ, which is the tuning parameter for LASSO, was selected for the smallest cross-validation error (λ_min). The final predictive models were refit using the significant components chosen by LASSO and λ_min. Calculations were performed on R using the Glmnet package using 489 radiomic features and 162 datasets, which had T1WI, T2WI, and Gd-T1WIs available with 10-fold outer-loop-cross-validation, which was repeated five times. Each patient was assigned as long-term or shot-term survivor according to the defined cut-off of overall survival for predicting long-term survivors and this cut-off was continuously changed which enables predicting modeling of long-term survivors at various thresholds. Although the fundamental aim of this analysis is the same as to that of the above-mentioned SPCA, the basic philosophy of analysis is different, as LASSO analysis only takes into account whether the patient lived longer or shorter than a given threshold while SPCA includes the whole survival duration into analysis.

## Results

**Figure 3:**
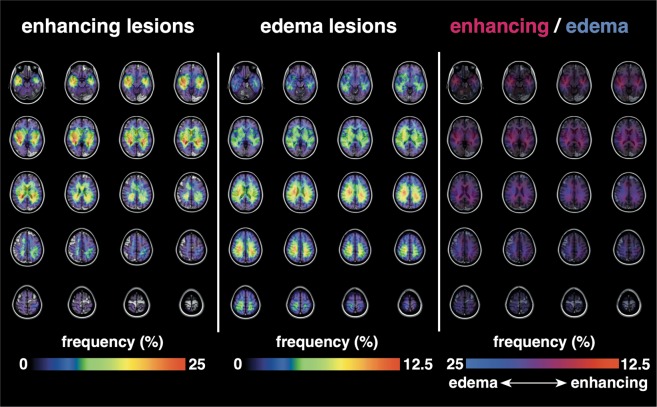
Lesion location mapping on the MNI152 standard brain atlas. Note that both enhancing and edema lesions were distributed symmetrically on both sides of the brain. Also note that enhancing lesions tended to occur in proximity to the ventricles compared with edema lesions.

One hundred one cases were *pMGMT*-met, and 100 were *MGMT* promotor unmethylated (*pMGMT*-unmet). A total of 489 texture features including first-order texture features, second-order features (Gray level co-occurrence matrix and Grey level run length matrix), and shape characteristics of the VOIs were extracted from 162 datasets, and location data from 182 datasets were collected. Location analysis showed that VOIs of core and edema accumulated symmetrically around the periventricular white matter (Fig. 3).

**Figure 4:**
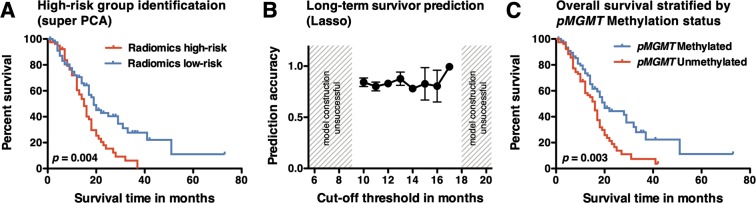
Kaplan-Meier curve of the cohort stratified by radiomic risk score **(A)** and *pMGMT* methylation status **(C)**. Both stratifications identified the poor-risk subgroup within the cohort. P values were calculated with the Log-rank test. LASSO was further used to predict long-term survivors using various cut-off in overall survival **(B)**. In this analysis, predictive modeling was possible only when the cut-off was set within 10 to 17 months.

**Figure 5:**
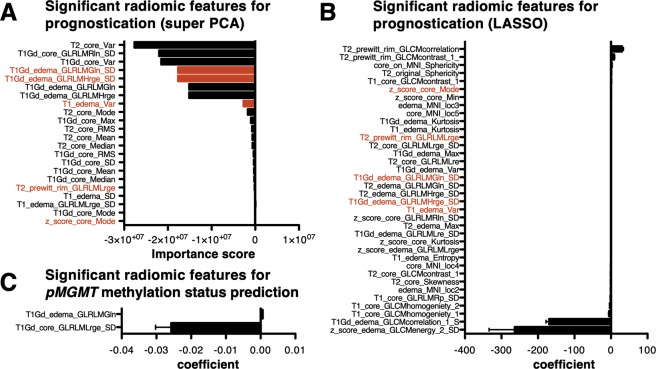
(**A**) Significant radiomic features corresponding to the prognostic outcome within the cohort using supervised principal component analysis. Twenty-two features were identified by supervised principal component analysis. (**B**) Significant radiomic features corresponding to the prognostic outcome within the cohort using LASSO. 36 features were identified by supervised principal component analysis. Items colored in “red” are those both identified by supervised principal component analysis and LASSO. (**C**) Two radiomic features were identified to be predictive of *pMGMT* methylation status. Of note, higher T1Gd_core_GLRLMLrge_SD was indicative of *pMGMT* unmethylated glioblastomas.

Next, prognostic modeling via radiomics was attempted by the aid of supervised principal component analysis, which was used to build a model capable of stratifying high- and low-risk GBM purely based on the initial MRI (Fig. 4A). The median overall survival time for radiomic high-risk GBM was 15 months, whereas that of radiomic low-risk GBM was 19 months (*p* = 0.004 Log-rank test). Furthermore, prediction of long-terms survivors at various overall survival cut-offs using LASSO also supported the finding by use of supervised principal component analysis. More specifically, predictions of patients surviving longer than 10, 11, 12, 13, 14, 15, 16 and 17 months were possible with high accuracy (Fig. 4B). These survival times are exactly when the Kaplan-Meier curves separates in Fig. 4A. As a reference, the median overall survival time of *pMGMT*-unmet GBM was 16 months, whereas that of *pMGMT*-met GBM was 20 months (*p* = 0.003 Log-rank test, Fig. 4C). The Supervised principal component analysis (SPCA) was able to identify 22 radiomic features that were significant for prognostication of GBM (Fig. 5A) and the trained prognostication model was able to label each subject into either a radiomic-high or -low risk class. Similarly, LASSO was able to identify 36 radiomic features that were significant for predicting patients surviving longer than 17 months (Fig. 5B). Five radiomic features were identified as prognostic features both by supervised principal component analysis and LASSO (Fig. 5A,B). Parametric survival analysis, however, using only these 5 radiomic features failed to converge to a meaningful predictive model for overall survival and univariate analysis revealed that only 3 among 5 features significantly correlated with survival duration of the patient (Supplementary Fig. 1). These results suggested that other listed radiomics factors were necessary for either SPCA or LASSO to construct their predictive model. On the other hand, attempt of predictive modeling of the *pMGMT*-met or unmet status via radiomics revealed to be unsatisfactory. Although radiomic feature selection predictive for *pMGMT* methylation status was pursued using LASSO regression (Fig. 5C), predictive accuracy of the *pMGMT* methylation status was as low as 67% on average measured by 10-fold cross-validation repeated five times (Table 1), suggesting limited use of this technique for *pMGMT* methylation status prediction. Univariate analysis revealed that age, pretreatment KPS, type of surgery, *pMGMT* methylation status and radiomic-risk stratification but not type of MRI used for analysis significantly correlated with overall survival (Fig. 4 and Supplementary Fig. 2). Cox regression analysis further revealed that age (*p* = 0.009), *pMGMT* methylation status (*p* = 0.001) and radiomic risk (*p* = 0.03) were independent prognostic factors. The hazard ratio of *pMGMT*-unmet was 2.04 (95% CI: 1.33–3.16, *p* = 0.001), and that of radiomic high-risk was 1.62 (95% CI: 1.04–2.52, *p = *0.03). This result also supports the conclusion that radiomic risk score and *pMGMT* methylation status are independent prognostic factors (Table 2). Aside from radiomic analysis being able to stratify poor and favorable patients in terms of overall survival, this was not the case in progression free survival (Supplementary Fig. 3).

**Table 1:** Prediction accuracy of *pMGMT* methylation status with radiomics.

	Average of 5 repetitive measures
Accuracy	67%
Sensitivity	67%
Specificity	66%
Positive predictive value	67%
Negative predictive value	67%
Prevalence of *pMGMT* methylation	50%

**Table 2:** Hazard ratio for overall survival of investigated factors.

Factor	Hazard ratio (lower to upper 95% CI)	*p* value
Age	1.02 (1.00–1.04)*	0.009**
Pretreatment KPS	0.99 (0.98–1.00)*	0.114
Type of Surgery	Partial to Total removal: 1.57 (0.97–2.51)	0.138
	Biopsy to Partial removal: 0.94 (0.50–1.71)	
	Biopsy to Total removal: 1.47 (0.81–2.61)	
*pMGMT* unmethylated	2.04 (1.33–3.16)	0.001**
Radiomic high-risk	1.62 (1.04–2.52)	0.031**

CI; confidence interval, *per unit change in regressor, **considered statistically significant.

**Figure 6:**
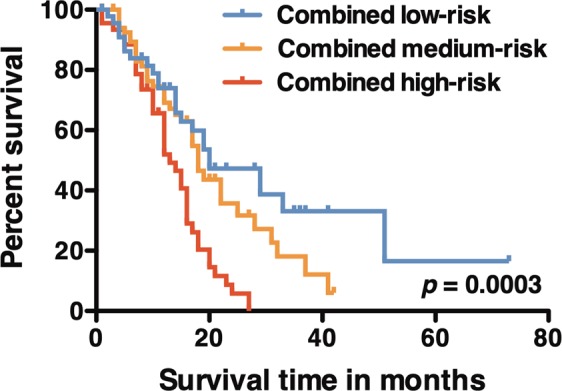
Kaplan-Meier curve of three different risk groups: low risk is composed of patients of radiomic low risk with *pMGMT* methylated status; medium risk is composed of patients of radiomic low risk with *pMGMT* unmethylated status or radiomic high risk with *pMGMT* methylated status; high risk is composed of patients of radiomic high risk with *pMGMT* unmethylated status. The P value was calculated with the Log-rank test.

Based on these results, we established a combined risk stratification using both radiomic risk and *pMGMT* methylation status of GBM. The cohort was categorized into three groups: 1. those with *pMGMT*-unmet and radiomic high risk considered high risk, 2. those with *pMGMT*-met and radiomic low risk considered low risk, and 3. the others considered intermediate risk. The median overall survival times for these groups were 13, 20, and 18 months, respectively (*p* = 0.0003, Fig. 6). The survival difference was mainly attributable to the high- versus medium- or low-risk group and the survival difference between low- and medium-risk group was not statically significant (*p* = 0.21).

## Discussion

Development of objective and high-throughput analysis of lesion textures enabled acquisition of quantitative imaging features on various imaging modalities, which is now termed radiomics^2–4,6,16^. The main goal of radiomics in neuro-oncology is to identify imaging biomarker(s) to predict biological features of brain tumors such as genetic status or clinical outcomes. Although this approach is potentially powerful for identifying *IDH* mutation status in high- and low-grade gliomas^6,11,17–19^, whether this method can be successfully applied for predicting *pMGMT* methylation status of GBM is still controversial. The current study is an extension of the authors previous study focusing on radiomics and WHO grade 2 and 3 gliomas^11^ and attempted to predict *pMGMT* methylation status of GBM by building a radiomic model and further stratify a poor prognostic subgroup of GBM patients based on radiomic profiles. A revised analytical system except for constructing the classifier was used in this study as the previous system was not yet incorporating secondary texture analysis^11^.

In radiomic analysis, intensity normalization is one of the key steps to ensure correct quantitative measurements of qualitative images such as MRI. Although there are other image intensity normalization methods which in some cases takes into account the tissue types within the image^2,20^, we have adopted the simplest but yet the closest to clinical practice. Similar to diagnostic radiologist thresholding and narrowing the window of images when reading, the current method performed similar thresholding of images to achieve intensity normalization for further radiomic analysis. In the current research, radiomic analysis consisted of location and texture analysis. Location analysis was included in the analysis to quantitatively evaluate the distribution of tumor locations, as this information could harbor valuable information about the biological features of GBM as reported previously^21,22^. The current system is unique in that contrast enhancing tumor core (VOI_core_) and the surrounding edematous lesions (VOI_edema_) were separately analyzed to retrieve as much radiomic information as possible.

Regarding the radiomic elements separating *pMGMT*-met from -unmet (Fig. 3), only two radiological features distinguished these two subgroups of GBM. Of note, second-order texture of the core lesion was heavily affected by the *pMGMT* methylation status. The sensitivity and selectivity, however, were not high enough to accurately predict *pMGMT* methylation status of GBM. Lesion location mapping also did not reveal any asymmetry in lesion occurrence between the two, a conclusion that is still controversial in the literature^16,21,22^. The present accuracy of 67% for predicting *pMGMT* methylation status is comparable to the accuracy described in previous investigations using different patient cohorts and analysis systems^16,23^. Those studies reported an accuracy ranging from 71 to 73%. Taking all these reports into consideration, the accuracy of radiomic prediction of *pMGMT* methylation status is at best around 70%. Considering that *pMGMT* methylation status is binary, this accuracy is far from satisfactory. Prediction of *pMGMT* methylation status may be limited by structural MRI alone.

On the other hand, when radiomics was compared with survival time of the cohort, radiomics was able to separate the cohort into poor versus good prognostic groups. As many as 68 among 162 cases (42%) were differently classified compared with the *pMGMT* methylation status. In fact, Cox regression analysis indeed identified that *pMGMT* methylation status and radiomic stratification were independent prognostic factors. It should further be noted that radiomic stratification was more prognostic than initial KPS or type of the surgery performed although residual tumor volume is now emerging as a key prognostic factor^24–26^. This finding is similar to that reported by Kickingereder *et al*. who used a different cohort and a different radiomic system and showed that radiomic risk scoring of GBM is independent of *pMGMT* methylation status^27^. It is also interesting that most of the radiomic features that the Supervised principal component analysis (SPCA) identified as prognostic derived from radiomic feature of the core lesions of GBM (14 out of 22). Although the reason in unclear, it is intuitively understandable that “enhancing core lesions” exhibit a more pronounce biological phenotype of the tumor than the surrounding edematous lesions. It is also of note that no location related information was identified as prognostic, which is different from our previous study focusing radiomic analysis and WHO grade 2 and 3 gliomas^11^. From a clinically practical point of view, as the SPCA model produces a “trained classifier object”, the actual numerical process of the predictive classification is not intuitive, which is difficult for clinician to incorporate this system into actual clinical practice. Furthermore, although the combination of *pMGMT* methylation status and radiomic risk score was able to identify an extremely high-risk group of patients (i.e. the combined high-risk group), the survival difference of combined low- and medium-risk was not statistically significant, requiring careful interpretation of the difference between these two groups.

Limitations of this study include the retrospective nature of the study and the absence of external cohort validation. It should also be pointed out that other machine learning methods for prognostication or *pMGMT* methylation status of GBM was not tested in this study and should be further pursued in future studies.

## Conclusion

The current study revealed that radiomics can be used to build a prognostic score stratifying high- and low-risk GBM; this score was an independent prognostic factor from *pMGMT* methylation status. On the other hand, predictive accuracy of *pMGMT* methylation status by radiomic analysis was 67%, which remains insufficient for practical use. A combination of radiomic score and *pMGMT* methylation status effectively provided a more accurate stratification of clinical outcomes for newly diagnosed GBM patients.

## Supplementary information

Supplementary figures

Supplementary Table

Supplementary Dataset

## Data Availability

Raw analyzed data are available in Supplementary Dataset.
