# Supplementary material for: Radiomics and MGMT promoter methylation for prognostication of newly diagnosed glioblastoma
Source: Sci Rep. 2019 Oct 8;9:14435. doi: 10.1038/s41598-019-50849-y (PMC6783410; doi:10.1038/s41598-019-50849-y)
Supplement: Supplementary file 1 — Supplementary figures [file 41598_2019_50849_MOESM1_ESM.pdf]

Supplementary figures

Radiomics and *MGMT* promoter methylation for prognostication of newly diagnosed glioblastoma

Takahiro Sasaki [1,2,3], Manabu Kinoshita [2,4,5*], Koji Fujita [2,3], Junya Fukai [2,3], Nobuhide Hayashi [1,2], Yuji Uematsu [2,3], Yoshiko Okita [2,6], Masahiro Nonaka [2,6,7], Shusuke Moriuchi [2,6,8], Takehiro Uda [2,9], Naohiro Tsuyuguchi [2,9,10], Hideyuki Arita [2,5], Kanji Mori [2,11], Kenichi Ishibashi [2,12], Koji Takano [2,13], Namiko Nishida [2,14], Tomoko Shofuda [2,15], Ema Yoshioka [2,15], Daisuke Kanematsu [2,16], Yoshinori Kodama [2,17], Masayuki Mano [2,18], Naoyuki Nakao [2,3], Yonehiro Kanemura [2,6,19]

[1]Department of Neurosurgery, Wakayama Rosai Hospital, Wakayama 640-8505, Japan, [2]Kansai Molecular Diagnosis Network for CNS Tumors, Osaka 540-0006, Japan, [3]Department of Neurosurgery, Wakayama Medical University, Wakayama 641-8509, Japan, [4]Department of Neurosurgery, Osaka International Cancer Institute, Osaka 541-8567, Japan, [5]Department of Neurosurgery, Osaka University Graduate School of Medicine, Suita 565-0871, Japan, [6]Department of Neurosurgery, Osaka National Hospital, National Hospital Organization, Osaka 540-0006, Japan, [7]Department of Neurosurgery, Kansai Medical University, Hirakata 573-1191, Japan, [8]Moriuchi Clinic of Neurosurgery, Izumiotsu, Osaka 595-0024, Japan, [9]Department of Neurosurgery, Osaka City University Graduate School of Medicine, Osaka 545-0051, Japan, [10]Department of Neurosurgery, Kindai University Faculty of Medicine, Sayama 589-8511, Japan, [11]Department of Neurosurgery, Kansai Rosai Hospital, Amagasaki 660-8511, Japan, [12]Department of Neurosurgery, Osaka City General Hospital, Osaka 534-0021, Japan, [13]Department of Neurosurgery, Toyonaka Municipal Hospital, Toyonaka 560-8565, Japan, [14]Department of Neurosurgery, Tazuke Kofukai Foundation, Medical Research Institute, Kitano Hospital, Osaka 530-8480, Japan, [15]Division of Stem Cell Research, Department of Biomedical Research and Innovation, Institute for Clinical Research, Osaka National Hospital, National Hospital Organization, Osaka 540-0006, Japan, [16]Division of Regenerative Medicine, Department of Biomedical Research and Innovation, Institute for Clinical Research, Osaka National Hospital, National Hospital Organization, Osaka 540-0006, Japan, [17]Department of Pathology and Applied Neurobiology, Graduate School of Medical Science, Kyoto Prefectural University of Medicine, Kyoto 602-8566, Japan, [18]Department of Pathology, Osaka National Hospital, National Hospital Organization, Osaka 540-0006, Japan, [19]Department of Biomedical Research and Innovation, Institute for Clinical Research, Osaka National Hospital, National Hospital Organization, Osaka 540-0006, Japan

Supplementary figure 1

Correlation of overall survival and 5 radiomic factors identified as significant both in supervised PCA and LASSO

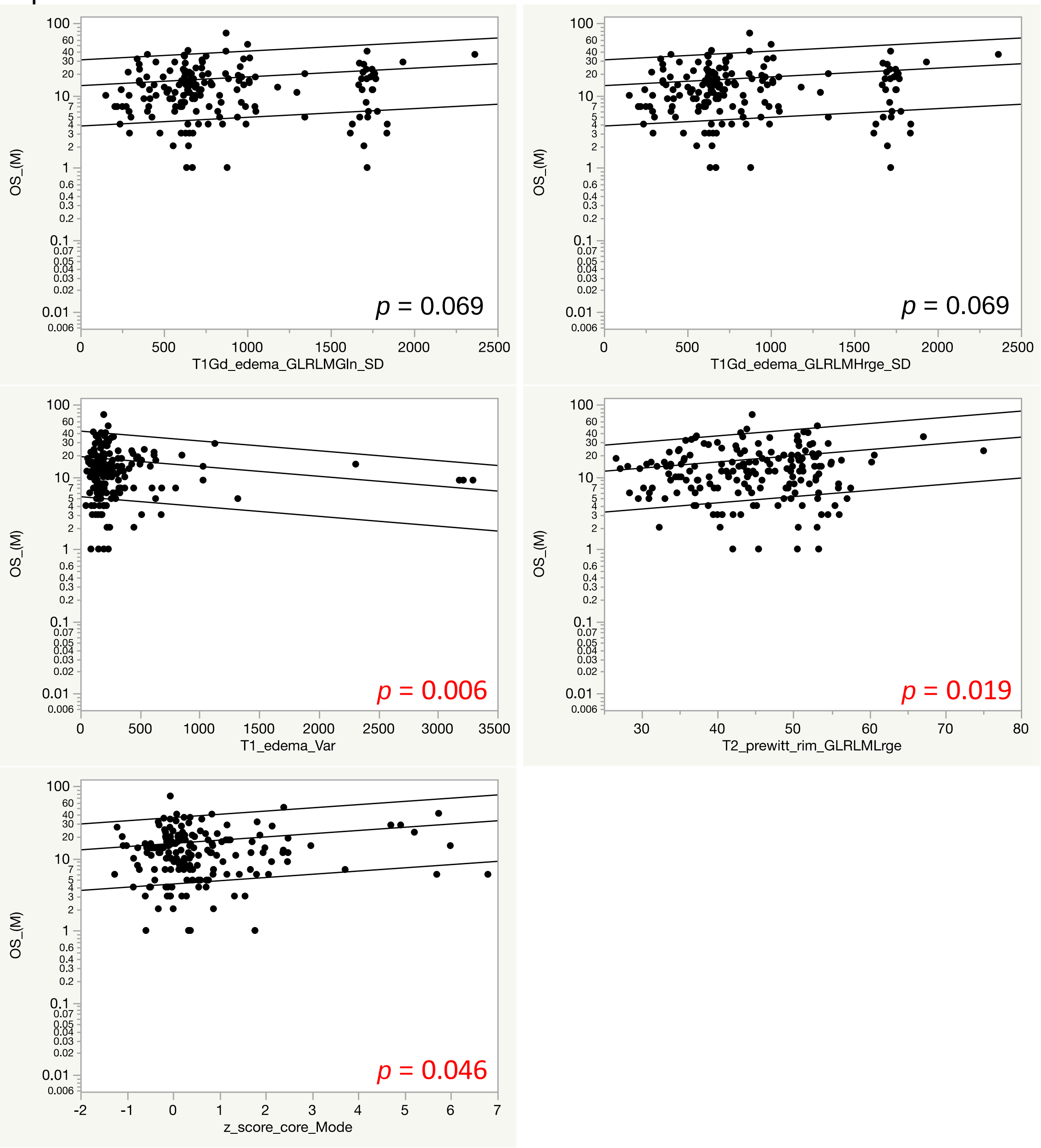

Univariate analysis of factors that were identified to be significant radiomic factors both in supervised PCA and LASSO was performed. As univariate predictors, only T1_edema_Var, T2_prewitt_rim_GLRLMLrge and z_score_core_Mode were identified as statistically significant. All *p* values were calculated by Wald Test.

Supplementary figure 2

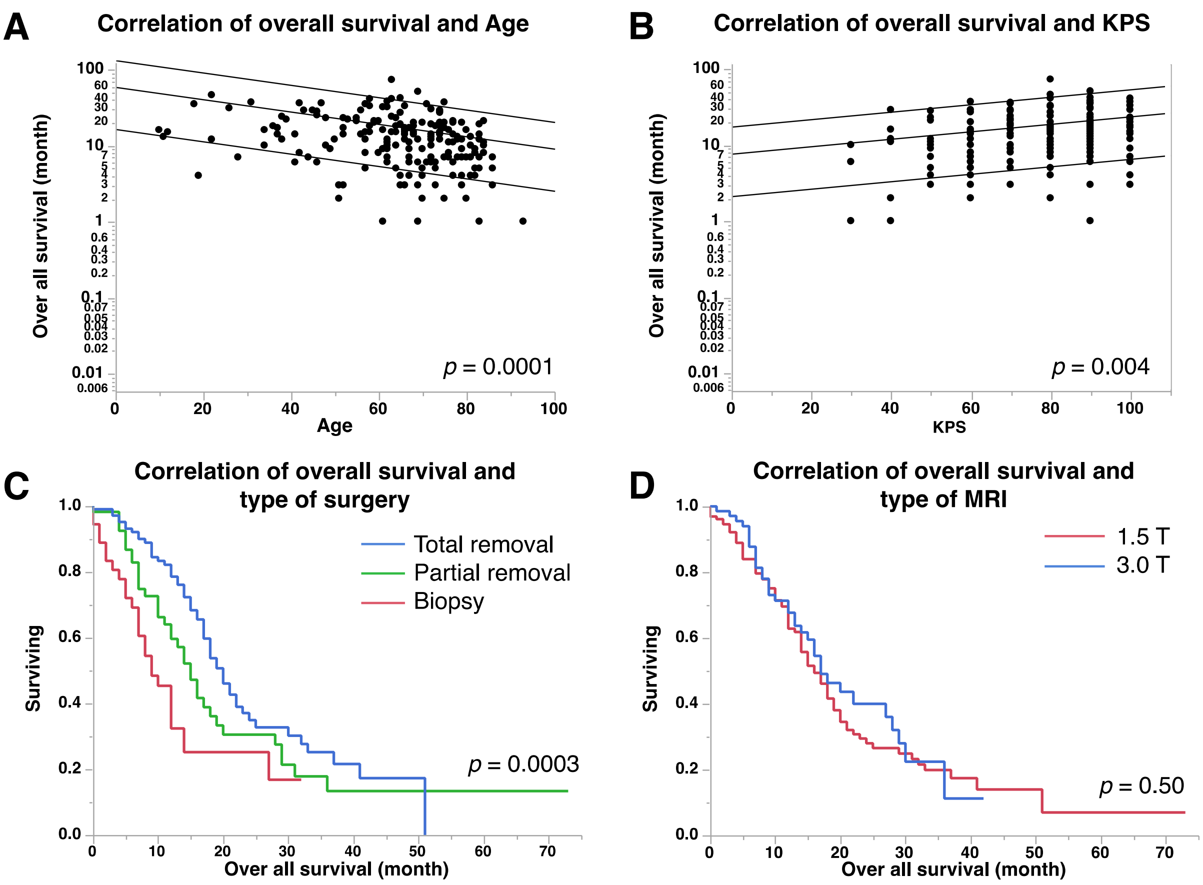

Univariate analysis of factors that potentially affect overall survival was performed. Both Age **(A)** and pretreatment KPS **(B)** significantly correlated with overall survival (p = 0.0001 and p = 0.004 respectively, Wald Test). Type of surgery **(C)** also affected overall survival (p = 0.0003, Log-Rank). Type of MRI used for radiomic analysis **(D)**, however, did not have any impact on overall survival (p = 0.50, Log-Rank).

Supplementary figure 3

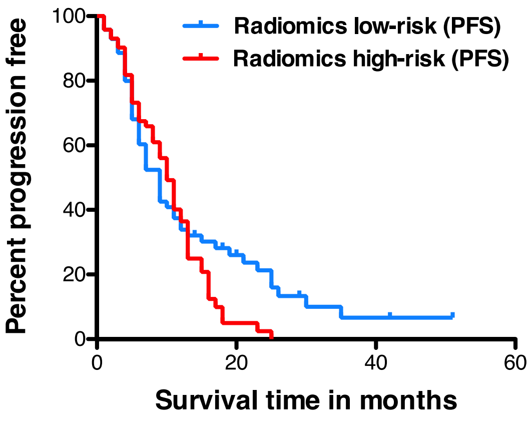

**Median survival**	
Radiomics low-risk (PFS)	9.000
Radiomics high-risk (PFS)	10.00
Ratio	0.9000
95% CI of ratio	0.2097 to 1.590

**Hazard Ratio**	
Ratio	0.7853
95% CI of ratio	0.5245 to 1.176

**Log-rank Test**	
P value	0.2407

/ Importance-score	Raw-score	Name /
[1,] "-138151.874"	"-2.192"	"T1Gd_core_Mode"
[2,] "-56722.813"	"-2.856"	"T2_core_Median"
[3,] "-55035.691"	"-2.541"	"T2_core_RMS"
[4,] "-54012.356"	"-2.551"	"T2_core_Mean"
[5,] "-44526.242"	"-2.873"	"T2_core_Max"
[6,] "-19501.782"	"-1.789"	"T2_edema_Max"
[7,] "-10286.364"	"-2.002"	"T2_core_GLRLMLre_SD"
[8,] "4125.706"	"1.738"	"T1_edema_GLRLMLrge_SD"
[9,] "1260.572"	"-1.885"	"T2_prewitt_rim_Kurtosis"

Radiomics risk stratification focusing on progression free survival was performed using the Superpc package in R. The threshold for constructing a survival prediction was searched by 10-fold outer-loop-cross-validation using Superpc and a threshold parameter of 1.65 was achieved as the best tuned parameter for the Supervised Principal Component Predictor model. Importance score of each radiomic feature was calculated with the threshold hold of Supervised Principal Component Predictor model set as 1.65, enabling visualization of significant radiomic features predictive of patient survival. Finally, a binary radiomic risk classification was achieved using the default and parameters as suggested in the Superpc reference manual of the superpc.predict function. More specifically, n.components of 1 and prediction.type of discrete were used with the threshold set to 1.65 as mentioned above. As can be appreciated in the Kaplan-Mayer survival curve, radiomics risk stratification was unsuccessful when progression free survival was used as endpoint. This was also supported by the fact that stratified two groups did not differ in Log-rank test.
