# Supplementary material for: Radiomics and MGMT promoter methylation for prognostication of newly diagnosed glioblastoma
Source: Sci Rep. 2019 Oct 8;9:14435. doi: 10.1038/s41598-019-50849-y (PMC6783410; doi:10.1038/s41598-019-50849-y)
Supplement: Supplementary file 2 — Supplementary Table [file 41598_2019_50849_MOESM2_ESM.pdf]

Supplementary Table

Radiomics and *MGMT* promoter methylation for prognostication of newly diagnosed glioblastoma

Takahiro Sasaki [1,2,3], Manabu Kinoshita [2,4,5*], Koji Fujita [2,3], Junya Fukai [2,3], Nobuhide Hayashi [1,2], Yuji Uematsu [2,3], Yoshiko Okita [2,6], Masahiro Nonaka [2,6,7], Shusuke Moriuchi [2,6,8], Takehiro Uda [2,9], Naohiro Tsuyuguchi [2,9,10], Hideyuki Arita [2,5], Kanji Mori [2,11], Kenichi Ishibashi [2,12], Koji Takano [2,13], Namiko Nishida [2,14], Tomoko Shofuda [2,15], Ema Yoshioka [2,15], Daisuke Kanematsu [2,16], Yoshinori Kodama [2,17], Masayuki Mano [2,18], Naoyuki Nakao [2,3], Yonehiro Kanemura [2,6,19]

[1]Department of Neurosurgery, Wakayama Rosai Hospital, Wakayama 640-8505, Japan, [2]Kansai Molecular Diagnosis Network for CNS Tumors, Osaka 540-0006, Japan, [3]Department of Neurosurgery, Wakayama Medical University, Wakayama 641-8509, Japan, [4]Department of Neurosurgery, Osaka International Cancer Institute, Osaka 541-8567, Japan, [5]Department of Neurosurgery, Osaka University Graduate School of Medicine, Suita 565-0871, Japan, [6]Department of Neurosurgery, Osaka National Hospital, National Hospital Organization, Osaka 540-0006, Japan, [7]Department of Neurosurgery, Kansai Medical University, Hirakata 573-1191, Japan, [8]Moriuchi Clinic of Neurosurgery, Izumiotsu, Osaka 595-0024, Japan, [9]Department of Neurosurgery, Osaka City University Graduate School of Medicine, Osaka 545-0051, Japan, [10]Department of Neurosurgery, Kindai University Faculty of Medicine, Sayama 589-8511, Japan, [11]Department of Neurosurgery, Kansai Rosai Hospital, Amagasaki 660-8511, Japan, [12]Department of Neurosurgery, Osaka City General Hospital, Osaka 534-0021, Japan, [13]Department of Neurosurgery, Toyonaka Municipal Hospital, Toyonaka 560-8565, Japan, [14]Department of Neurosurgery, Tazuke Kofukai Foundation, Medical Research Institute, Kitano Hospital, Osaka 530-8480, Japan, [15]Division of Stem Cell Research, Department of Biomedical Research and Innovation, Institute for Clinical Research, Osaka National Hospital, National Hospital Organization, Osaka 540-0006, Japan, [16]Division of Regenerative Medicine, Department of Biomedical Research and Innovation, Institute for Clinical Research, Osaka National Hospital, National Hospital Organization, Osaka 540-0006, Japan, [17]Department of Pathology and Applied Neurobiology, Graduate School of Medical Science, Kyoto Prefectural University of Medicine, Kyoto 602-8566, Japan, [18]Department of Pathology, Osaka National Hospital, National Hospital Organization, Osaka 540-0006, Japan, [19]Department of Biomedical Research and Innovation, Institute for Clinical Research, Osaka National Hospital, National Hospital Organization, Osaka 540-0006, Japan

	Radiomic designation code	Contents of values
1	T1Gd_original_Total_Surface_Area	Total surface area (A) of T1Gd area.
2	T1Gd_original_Total_Volume	Total volume (V) of T1Gd area.
3	T1Gd_original_Compactness01	Value calculated by the following equation of T1Gd area; $compactness1 = \frac{V}{\sqrt{\pi} * A^{\frac{2}{3}}}$
4	T1Gd_original_Compactness02	Value calculated by the following equation of T1Gd area; $compactness2 = 36\pi \frac{A^2}{V^3}$
5	T1Gd_original_Spherical_Disporoportion	Value calculated by the following equation of T1Gd area; $spherical\_disproportion = \frac{A}{4\pi * R^2} = \frac{A}{(6\sqrt{\pi} * V)^{\frac{2}{3}}}$
6	T1Gd_original_Sphericity	Value calculated by the following equation of T1Gd area; $sphericity = \frac{(6\pi^2 V)^{\frac{2}{3}}}{A}$
7	T1Gd_original_Surface_to_Volume_ratio	Value calculated by the following equation of T1Gd area; $surface\_volume\_ratio = \frac{A}{V}$
8	T2_original_Total_Surface_Area	Total surface area (A) of T2 area.
9	T2_original_Total_Volume	Total volume (V) of T2 area.
10	T2_original_Compactness01	Value calculated by the following equation of T2 area; $compactness1 = \frac{V}{\sqrt{\pi} * A^{\frac{2}{3}}}$
11	T2_original_Compactness02	Value calculated by the following equation of T2 area; $compactness2 = 36\pi \frac{A^2}{V^3}$
12	T2_original_Spherical_Disporoportion	Value calculated by the following equation of T2 area; $spherical\_disproportion = \frac{A}{4\pi * R^2} = \frac{A}{(6\sqrt{\pi} * V)^{\frac{2}{3}}}$
13	T2_original_Sphericity	Value calculated by the following equation of T2 area; $sphericity = \frac{(6\pi^2 V)^{\frac{2}{3}}}{A}$

14	T2_original_Surface_to_Volume_ratio	Value calculated by the following equation of T2 area; $surface\_volume\_ratio = \frac{A}{V}$
15	core_MNI_loc0	Occupancy rate of area “0” multiplied by 1 of the MNI structural atlas within the core VOI. This area represents white matter.
16	core_MNI_loc1	Occupancy rate of area “1” multiplied by 2 of the MNI structural atlas within the core VOI. This area represents lateral ventricles.
17	core_MNI_loc2	Occupancy rate of area “2” multiplied by 3 of the MNI structural atlas within the core VOI. This area represents the cerebrum.
18	core_MNI_loc3	Occupancy rate of area “3” multiplied by 4 of the MNI structural atlas within the core VOI. This area represents the frontal lobe.
19	core_MNI_loc4	Occupancy rate of area “4” multiplied by 5 of the MNI structural atlas within the core VOI. This area represents the insular lobe.
20	core_MNI_loc5	Occupancy rate of area “5” multiplied by 6 of the MNI structural atlas within the core VOI. This area represents the occipital lobe.
21	core_MNI_loc6	Occupancy rate of area “6” multiplied by 7 of the MNI structural atlas within the core VOI. This area represents the parietal lobe.
22	core_MNI_loc7	Occupancy rate of area “7” multiplied by 8 of the MNI structural atlas within the core VOI. This area represents the basal ganglia.
23	core_MNI_loc8	Occupancy rate of area “8” multiplied by 9 of the MNI structural atlas within the core VOI. This area represents the temporal lobe.
24	core_MNI_loc9	Occupancy rate of area “9” multiplied by 10 of the MNI structural atlas within the core VOI. This area represents the thalamus.
25	edema_MNI_loc0	Occupancy rate of area “0” multiplied by 1 of the MNI structural atlas within the edema VOI. This area represents white matter.
26	edema_MNI_loc1	Occupancy rate of area “1” multiplied by 2 of the MNI structural atlas within the edema VOI. This area represents lateral ventricles.
27	edema_MNI_loc2	Occupancy rate of area “2” multiplied by 3 of the MNI structural atlas within the edema VOI. This area represents the cerebrum.
28	edema_MNI_loc3	Occupancy rate of area “3” multiplied by 4 of the MNI structural atlas within the edema VOI. This area represents the frontal lobe.
29	edema_MNI_loc4	Occupancy rate of area “4” multiplied by 5 of the MNI structural atlas within the edema VOI. This area represents the insular lobe.
30	edema_MNI_loc5	Occupancy rate of area “5” multiplied by 6 of the MNI structural atlas within the edema VOI. This area represents the occipital lobe.
31	edema_MNI_loc6	Occupancy rate of area “6” multiplied by 7 of the MNI structural atlas within the edema VOI. This area represents the parietal lobe.
32	edema_MNI_loc7	Occupancy rate of area “7” multiplied by 8 of the MNI structural atlas within the edema VOI. This area represents the basal ganglia.
33	edema_MNI_loc8	Occupancy rate of area “8” multiplied by 9 of the MNI structural atlas within the edema VOI. This area represents the temporal lobe.

34	edema_MNI_loc9	Occupancy rate of area “9” multiplied by 10 of the MNI structural atlas within the edema VOI. This area represents the thalamus.
35	T1Gd_core_Mean	Mean of VOI_core in 256-leveled Gadolinium enhanced T1-weighted image.
36	T1Gd_core_SD	Standard deviation of VOI_core in 256-leveled Gadolinium enhanced T1-weighted image.
37	T1Gd_core_Var	Variance of VOI_core in 256-leveled Gadolinium enhanced T1-weighted image.
38	T1Gd_core_RMS	Root Mean Square of VOI_core in 256-leveled Gadolinium enhanced T1-weighted image.
39	T1Gd_core_Max	Maximum of VOI_core in 256-leveled Gadolinium enhanced T1-weighted image.
40	T1Gd_core_Min	Minimum of VOI_core in 256-leveled Gadolinium enhanced T1-weighted image.
41	T1Gd_core_Median	Median of VOI_core in 256-leveled Gadolinium enhanced T1-weighted image.
42	T1Gd_core_Mode	Mode of VOI_core in 256-leveled Gadolinium enhanced T1-weighted image.
43	T1Gd_core_Entropy	Entropy of VOI_core in 256-leveled Gadolinium enhanced T1-weighted image.
44	T1Gd_core_Kurtosis	Kurtosis of VOI_core in 256-leveled Gadolinium enhanced T1-weighted image.
45	T1Gd_core_Skewness	Skewness of VOI_core in 256-leveled Gadolinium enhanced T1-weighted image.
46	T1Gd_core_GLCMcontrast_1	Mean contrast of GLCM in VOI_core in 256-leveled Gadolinium enhanced T1-weighted image with offset set to 1.
47	T1Gd_core_GLCMcontrast_2	Mean contrast of GLCM in VOI_core in 256-leveled Gadolinium enhanced T1-weighted image with offset set to 2.
48	T1Gd_core_GLCMcontrast_3	Mean contrast of GLCM in VOI_core in 256-leveled Gadolinium enhanced T1-weighted image with offset set to 3.
49	T1Gd_core_GLCMenergy_1	Mean energy of GLCM in VOI_core in 256-leveled Gadolinium enhanced T1-weighted image with offset set to 1.
50	T1Gd_core_GLCMenergy_2	Mean energy of GLCM in VOI_core in 256-leveled Gadolinium enhanced T1-weighted image with offset set to 2.
51	T1Gd_core_GLCMenergy_3	Mean energy of GLCM in VOI_core in 256-leveled Gadolinium enhanced T1-weighted image with offset set to 3.
52	T1Gd_core_GLCMhomogeniety_1	Mean homogeneity of GLCM in VOI_core in 256-leveled Gadolinium enhanced T1-weighted image with offset set to 1.
53	T1Gd_core_GLCMhomogeniety_2	Mean homogeneity of GLCM in VOI_core in 256-leveled Gadolinium enhanced T1-weighted image with offset set to 2.
54	T1Gd_core_GLCMhomogeniety_3	Mean homogeneity of GLCM in VOI_core in 256-leveled Gadolinium enhanced T1-weighted image with offset set to 3.
55	T1Gd_core_GLRLMSre	Short run emphasis of GLRLM in VOI_core in 256-leveled Gadolinium enhanced T1-weighted image.
56	T1Gd_core_GLRLMLre	Long run emphasis of GLRLM in VOI_core in 256-leveled Gadolinium enhanced T1-weighted image.
57	T1Gd_core_GLRLMGln	GRAY LEVEL NON-UNIFORMITY of GLRLM in VOI_core in 256-leveled Gadolinium enhanced T1-weighted image.
58	T1Gd_core_GLRLMRp	RUN PERCENTAGE of GLRLM in VOI_core in 256-leveled Gadolinium enhanced T1-weighted image.

59	T1Gd_core_GLRLMRln	RUN LENGTH NON-UNIFORMITY in VOI_core in 256-leveled Gadolinium enhanced T1-weighted image.
60	T1Gd_core_GLRLMLrge	LOW GRAY LEVEL RUN EMPHASIS in VOI_core in 256-leveled Gadolinium enhanced T1-weighted image.
61	T1Gd_core_GLRLMHrge	HIGH GRAY LEVEL RUN EMPHASIS in VOI_core in 256-leveled Gadolinium enhanced T1-weighted image.
62	T1Gd_core_GLCMcontrast_1_SD	Standard deviation of contrast of GLCM in VOI_core in 256-leveled Gadolinium enhanced T1-weighted image with offset set to 1.
63	T1Gd_core_GLCMcontrast_2_SD	Standard deviation of contrast of GLCM in VOI_core in 256-leveled Gadolinium enhanced T1-weighted image with offset set to 2.
64	T1Gd_core_GLCMcontrast_3_SD	Standard deviation of contrast of GLCM in VOI_core in 256-leveled Gadolinium enhanced T1-weighted image with offset set to 3.
65	T1Gd_core_GLCMenergy_1_SD	Standard deviation of energy of GLCM in VOI_core in 256-leveled Gadolinium enhanced T1-weighted image with offset set to 1.
66	T1Gd_core_GLCMenergy_2_SD	Standard deviation of energy of GLCM in VOI_core in 256-leveled Gadolinium enhanced T1-weighted image with offset set to 2.
67	T1Gd_core_GLCMenergy_3_SD	Standard deviation of energy of GLCM in VOI_core in 256-leveled Gadolinium enhanced T1-weighted image with offset set to 3.
68	T1Gd_core_GLCMhomogeniety_1_SD	Standard deviation of homogeneity of GLCM in VOI_core in 256-leveled Gadolinium enhanced T1-weighted image with offset set to 1.
69	T1Gd_core_GLCMhomogeniety_2_SD	Standard deviation of homogeneity of GLCM in VOI_core in 256-leveled Gadolinium enhanced T1-weighted image with offset set to 2.
70	T1Gd_core_GLCMhomogeniety_3_SD	Standard deviation of homogeneity of GLCM in VOI_core in 256-leveled Gadolinium enhanced T1-weighted image with offset set to 3.
71	T1Gd_core_GLRLMSre_SD	Standard deviation of Short run emphasis of GLRLM in VOI_core in 256-leveled Gadolinium enhanced T1-weighted image.
72	T1Gd_core_GLRLMLre_SD	Standard deviation of Long run emphasis of GLRLM in VOI_core in 256-leveled Gadolinium enhanced T1-weighted image.
73	T1Gd_core_GLRLMGln_SD	Standard deviation of GRAY LEVEL NON-UNIFORMITY of GLRLM in VOI_core in 256-leveled Gadolinium enhanced T1-weighted image.
74	T1Gd_core_GLRLMRp_SD	Standard deviation of RUN PERCENTAGE of GLRLM in VOI_core in 256-leveled Gadolinium enhanced T1-weighted image.
75	T1Gd_core_GLRLMRln_SD	Standard deviation of RUN LENGTH NON-UNIFORMITY in VOI_core in 256-leveled Gadolinium enhanced T1-weighted image.
76	T1Gd_core_GLRLMLrge_SD	Standard deviation of LOW GRAY LEVEL RUN EMPHASIS in VOI_core in 256-leveled Gadolinium enhanced T1-weighted image.
77	T1Gd_core_GLRLMHrge_SD	Standard deviation of HIGH GRAY LEVEL RUN EMPHASIS in VOI_core in 256-leveled Gadolinium enhanced T1-weighted image.
78	T1Gd_edema_Mean	Mean of VOI_edema in 256-leveled Gadolinium enhanced T1-weighted image.
79	T1Gd_edema_SD	Standard deviation of VOI_edema in 256-leveled Gadolinium enhanced T1-weighted image.
80	T1Gd_edema_Var	Variance of VOI_edema in 256-leveled Gadolinium enhanced T1-weighted image.

81	T1Gd_edema_RMS	Root Mean Square of VOI_edema in 256-leveled Gadolinium enhanced T1-weighted image.
82	T1Gd_edema_Max	Maximum of VOI_edema in 256-leveled Gadolinium enhanced T1-weighted image.
83	T1Gd_edema_Min	Minimum of VOI_edema in 256-leveled Gadolinium enhanced T1-weighted image.
84	T1Gd_edema_Median	Median of VOI_edema in 256-leveled Gadolinium enhanced T1-weighted image.
85	T1Gd_edema_Mode	Mode of VOI_edema in 256-leveled Gadolinium enhanced T1-weighted image.
86	T1Gd_edema_Entropy	Entropy of VOI_edema in 256-leveled Gadolinium enhanced T1-weighted image.
87	T1Gd_edema_Kurtosis	Kurtosis of VOI_edema in 256-leveled Gadolinium enhanced T1-weighted image.
88	T1Gd_edema_Skewness	Skewness of VOI_edema in 256-leveled Gadolinium enhanced T1-weighted image.
89	T1Gd_edema_GLCMcontrast_1	Mean contrast of GLCM in VOI_edema in 256-leveled Gadolinium enhanced T1-weighted image with offset set to 1.
90	T1Gd_edema_GLCMcontrast_2	Mean contrast of GLCM in VOI_edema in 256-leveled Gadolinium enhanced T1-weighted image with offset set to 2.
91	T1Gd_edema_GLCMcontrast_3	Mean contrast of GLCM in VOI_edema in 256-leveled Gadolinium enhanced T1-weighted image with offset set to 3.
92	T1Gd_edema_GLCMenergy_1	Mean energy of GLCM in VOI_edema in 256-leveled Gadolinium enhanced T1-weighted image with offset set to 1.
93	T1Gd_edema_GLCMenergy_2	Mean energy of GLCM in VOI_edema in 256-leveled Gadolinium enhanced T1-weighted image with offset set to 2.
94	T1Gd_edema_GLCMenergy_3	Mean energy of GLCM in VOI_edema in 256-leveled Gadolinium enhanced T1-weighted image with offset set to 3.
95	T1Gd_edema_GLCMhomogeniety_1	Mean homogeneity of GLCM in VOI_edema in 256-leveled Gadolinium enhanced T1-weighted image with offset set to 1.
96	T1Gd_edema_GLCMhomogeniety_2	Mean homogeneity of GLCM in VOI_edema in 256-leveled Gadolinium enhanced T1-weighted image with offset set to 2.
97	T1Gd_edema_GLCMhomogeniety_3	Mean homogeneity of GLCM in VOI_edema in 256-leveled Gadolinium enhanced T1-weighted image with offset set to 3.
98	T1Gd_edema_GLRLMSre	Short run emphasis of GLRLM in VOI_edema in 256-leveled Gadolinium enhanced T1-weighted image.
99	T1Gd_edema_GLRLMLre	Long run emphasis of GLRLM in VOI_edema in 256-leveled Gadolinium enhanced T1-weighted image.
100	T1Gd_edema_GLRLMGln	GRAY LEVEL NON-UNIFORMITY of GLRLM in VOI_edema in 256-leveled Gadolinium enhanced T1-weighted image.
101	T1Gd_edema_GLRLMRp	RUN PERCENTAGE of GLRLM in VOI_edema in 256-leveled Gadolinium enhanced T1-weighted image.
102	T1Gd_edema_GLRLMRln	RUN LENGTH NON-UNIFORMITY in VOI_edema in 256-leveled Gadolinium enhanced T1-weighted image.
103	T1Gd_edema_GLRLMLrge	LOW GRAY LEVEL RUN EMPHASIS in VOI_edema in 256-leveled Gadolinium enhanced T1-weighted image.
104	T1Gd_edema_GLRLMHrge	HIGH GRAY LEVEL RUN EMPHASIS in VOI_edema in 256-leveled Gadolinium enhanced T1-weighted image.

105	T1Gd_edema_GLCMcontrast_1_SD	Standard deviation of contrast of GLCM in VOI_edema in 256-leveled Gadolinium enhanced T1-weighted image with offset set to 1.
106	T1Gd_edema_GLCMcontrast_2_SD	Standard deviation of contrast of GLCM in VOI_edema in 256-leveled Gadolinium enhanced T1-weighted image with offset set to 2.
107	T1Gd_edema_GLCMcontrast_3_SD	Standard deviation of contrast of GLCM in VOI_edema in 256-leveled Gadolinium enhanced T1-weighted image with offset set to 3.
108	T1Gd_edema_GLCMenergy_1_SD	Standard deviation of energy of GLCM in VOI_edema in 256-leveled Gadolinium enhanced T1-weighted image with offset set to 1.
109	T1Gd_edema_GLCMenergy_2_SD	Standard deviation of energy of GLCM in VOI_edema in 256-leveled Gadolinium enhanced T1-weighted image with offset set to 2.
110	T1Gd_edema_GLCMenergy_3_SD	Standard deviation of energy of GLCM in VOI_edema in 256-leveled Gadolinium enhanced T1-weighted image with offset set to 3.
111	T1Gd_edema_GLCMhomogeniety_1_SD	Standard deviation of homogeneity of GLCM in VOI_edema in 256-leveled Gadolinium enhanced T1-weighted image with offset set to 1.
112	T1Gd_edema_GLCMhomogeniety_2_SD	Standard deviation of homogeneity of GLCM in VOI_edema in 256-leveled Gadolinium enhanced T1-weighted image with offset set to 2.
113	T1Gd_edema_GLCMhomogeniety_3_SD	Standard deviation of homogeneity of GLCM in VOI_edema in 256-leveled Gadolinium enhanced T1-weighted image with offset set to 3.
114	T1Gd_edema_GLRLMSre_SD	Standard deviation of Short run emphasis of GLRLM in VOI_edema in 256-leveled Gadolinium enhanced T1-weighted image.
115	T1Gd_edema_GLRLMLre_SD	Standard deviation of Long run emphasis of GLRLM in VOI_edema in 256-leveled Gadolinium enhanced T1-weighted image.
116	T1Gd_edema_GLRLMGln_SD	Standard deviation of GRAY LEVEL NON-UNIFORMITY of GLRLM in VOI_edema in 256-leveled Gadolinium enhanced T1-weighted image.
117	T1Gd_edema_GLRLMRp_SD	Standard deviation of RUN PERCENTAGE of GLRLM in VOI_edema in 256-leveled Gadolinium enhanced T1-weighted image.
118	T1Gd_edema_GLRLMRln_SD	Standard deviation of RUN LENGTH NON-UNIFORMITY in VOI_edema in 256-leveled Gadolinium enhanced T1-weighted image.
119	T1Gd_edema_GLRLMLrge_SD	Standard deviation of LOW GRAY LEVEL RUN EMPHASIS in VOI_edema in 256-leveled Gadolinium enhanced T1-weighted image.
120	T1Gd_edema_GLRLMHrge_SD	Standard deviation of HIGH GRAY LEVEL RUN EMPHASIS in VOI_edema in 256-leveled Gadolinium enhanced T1-weighted image.
121	T2_core_Mean	Mean of VOI_core in 256-leveled T2-weighted image.
122	T2_core_SD	Standard deviation of VOI_core in 256-leveled T2-weighted image.
123	T2_core_Var	Variance of VOI_core in 256-leveled T2-weighted image.
124	T2_core_RMS	Root Mean Square of VOI_core in 256-leveled T2-weighted image.
125	T2_core_Max	Maximum of VOI_core in 256-leveled T2-weighted image.
126	T2_core_Min	Minimum of VOI_core in 256-leveled T2-weighted image.

127	T2_core_Median	Median of VOI_core in 256-leveled T2-weighted image.
128	T2_core_Mode	Mode of VOI_core in 256-leveled T2-weighted image.
129	T2_core_Entropy	Entropy of VOI_core in 256-leveled T2-weighted image.
130	T2_core_Kurtosis	Kurtosis of VOI_core in 256-leveled T2-weighted image.
131	T2_core_Skewness	Skewness of VOI_core in 256-leveled T2-weighted image.
132	T2_core_GLCMcontrast_1	Mean contrast of GLCM in VOI_core in 256-leveled T2-weighted image with offset set to 1.
133	T2_core_GLCMcontrast_2	Mean contrast of GLCM in VOI_core in 256-leveled T2-weighted image with offset set to 2.
134	T2_core_GLCMcontrast_3	Mean contrast of GLCM in VOI_core in 256-leveled T2-weighted image with offset set to 3.
135	T2_core_GLCMenergy_1	Mean energy of GLCM in VOI_core in 256-leveled T2-weighted image with offset set to 1.
136	T2_core_GLCMenergy_2	Mean energy of GLCM in VOI_core in 256-leveled T2-weighted image with offset set to 2.
137	T2_core_GLCMenergy_3	Mean energy of GLCM in VOI_core in 256-leveled T2-weighted image with offset set to 3.
138	T2_core_GLCMhomogeniety_1	Mean homogeneity of GLCM in VOI_core in 256-leveled T2-weighted image with offset set to 1.
139	T2_core_GLCMhomogeniety_2	Mean homogeneity of GLCM in VOI_core in 256-leveled T2-weighted image with offset set to 2.
140	T2_core_GLCMhomogeniety_3	Mean homogeneity of GLCM in VOI_core in 256-leveled T2-weighted image with offset set to 3.
141	T2_core_GLRLMSre	Short run emphasis of GLRLM in VOI_core in 256-leveled T2-weighted image.
142	T2_core_GLRLMLre	Long run emphasis of GLRLM in VOI_core in 256-leveled T2-weighted image.
143	T2_core_GLRLMGln	GRAY LEVEL NON-UNIFORMITY of GLRLM in VOI_core in 256-leveled T2-weighted image.
144	T2_core_GLRLMRp	RUN PERCENTAGE of GLRLM in VOI_core in 256-leveled T2-weighted image.
145	T2_core_GLRLMRln	RUN LENGTH NON-UNIFORMITY in VOI_core in 256-leveled T2-weighted image.
146	T2_core_GLRLMLrge	LOW GRAY LEVEL RUN EMPHASIS in VOI_core in 256-leveled T2-weighted image.
147	T2_core_GLRLMHrge	HIGH GRAY LEVEL RUN EMPHASIS in VOI_core in 256-leveled T2-weighted image.
148	T2_core_GLCMcontrast_1_SD	Standard deviation of contrast of GLCM in VOI_core in 256-leveled T2-weighted image with offset set to 1.
149	T2_core_GLCMcontrast_2_SD	Standard deviation of contrast of GLCM in VOI_core in 256-leveled T2-weighted image with offset set to 2.
150	T2_core_GLCMcontrast_3_SD	Standard deviation of contrast of GLCM in VOI_core in 256-leveled T2-weighted image with offset set to 3.
151	T2_core_GLCMenergy_1_SD	Standard deviation of energy of GLCM in VOI_core in 256-leveled T2-weighted image with offset set to 1.
152	T2_core_GLCMenergy_2_SD	Standard deviation of energy of GLCM in VOI_core in 256-leveled T2-weighted image with offset set to 2.
153	T2_core_GLCMenergy_3_SD	Standard deviation of energy of GLCM in VOI_core in 256-leveled T2-weighted image with offset set to 3.

154	T2_core_GLCMhomogeniety_1_SD	Standard deviation of homogeneity of GLCM in VOI_core in 256-leveled T2-weighted image with offset set to 1.
155	T2_core_GLCMhomogeniety_2_SD	Standard deviation of homogeneity of GLCM in VOI_core in 256-leveled T2-weighted image with offset set to 2.
156	T2_core_GLCMhomogeniety_3_SD	Standard deviation of homogeneity of GLCM in VOI_core in 256-leveled T2-weighted image with offset set to 3.
157	T2_core_GLRLMSre_SD	Standard deviation of Short run emphasis of GLRLM in VOI_core in 256-leveled T2-weighted image.
158	T2_core_GLRLMLre_SD	Standard deviation of Long run emphasis of GLRLM in VOI_core in 256-leveled T2-weighted image.
159	T2_core_GLRLMGln_SD	Standard deviation of GRAY LEVEL NON-UNIFORMITY of GLRLM in VOI_core in 256-leveled T2-weighted image.
160	T2_core_GLRLMRp_SD	Standard deviation of RUN PERCENTAGE of GLRLM in VOI_core in 256-leveled T2-weighted image.
161	T2_core_GLRLMRln_SD	Standard deviation of RUN LENGTH NON-UNIFORMITY in VOI_core in 256-leveled T2-weighted image.
162	T2_core_GLRLMLrge_SD	Standard deviation of LOW GRAY LEVEL RUN EMPHASIS in VOI_core in 256-leveled T2-weighted image.
163	T2_core_GLRLMHrge_SD	Standard deviation of HIGH GRAY LEVEL RUN EMPHASIS in VOI_core in 256-leveled T2-weighted image.
164	T2_edema_Mean	Mean of VOI_edema in 256-leveled T2-weighted image.
165	T2_edema_SD	Standard deviation of VOI_edema in 256-leveled T2-weighted image.
166	T2_edema_Var	Variance of VOI_edema in 256-leveled T2-weighted image.
167	T2_edema_RMS	Root Mean Square of VOI_edema in 256-leveled T2-weighted image.
168	T2_edema_Max	Maximum of VOI_edema in 256-leveled T2-weighted image.
169	T2_edema_Min	Minimum of VOI_edema in 256-leveled T2-weighted image.
170	T2_edema_Median	Median of VOI_edema in 256-leveled T2-weighted image.
171	T2_edema_Mode	Mode of VOI_edema in 256-leveled T2-weighted image.
172	T2_edema_Entropy	Entropy of VOI_edema in 256-leveled T2-weighted image.
173	T2_edema_Kurtosis	Kurtosis of VOI_edema in 256-leveled T2-weighted image.
174	T2_edema_Skewness	Skewness of VOI_edema in 256-leveled T2-weighted image.
175	T2_edema_GLCMcontrast_1	Mean contrast of GLCM in VOI_edema in 256-leveled T2-weighted image with offset set to 1.
176	T2_edema_GLCMcontrast_2	Mean contrast of GLCM in VOI_edema in 256-leveled T2-weighted image with offset set to 2.
177	T2_edema_GLCMcontrast_3	Mean contrast of GLCM in VOI_edema in 256-leveled T2-weighted image with offset set to 3.
178	T2_edema_GLCMenergy_1	Mean energy of GLCM in VOI_edema in 256-leveled T2-weighted image with offset set to 1.
179	T2_edema_GLCMenergy_2	Mean energy of GLCM in VOI_edema in 256-leveled T2-weighted image with offset set to 2.
180	T2_edema_GLCMenergy_3	Mean energy of GLCM in VOI_edema in 256-leveled T2-weighted image with offset set to 3.

181	T2_edema_GLCMhomogeniety_1	Mean homogeneity of GLCM in VOI_edema in 256-leveled T2-weighted image with offset set to 1.
182	T2_edema_GLCMhomogeniety_2	Mean homogeneity of GLCM in VOI_edema in 256-leveled T2-weighted image with offset set to 2.
183	T2_edema_GLCMhomogeniety_3	Mean homogeneity of GLCM in VOI_edema in 256-leveled T2-weighted image with offset set to 3.
184	T2_edema_GLRLMSre	Short run emphasis of GLRLM in VOI_edema in 256-leveled T2-weighted image.
185	T2_edema_GLRLMLre	Long run emphasis of GLRLM in VOI_edema in 256-leveled T2-weighted image.
186	T2_edema_GLRLMGln	GRAY LEVEL NON-UNIFORMITY of GLRLM in VOI_edema in 256-leveled T2-weighted image.
187	T2_edema_GLRLMRp	RUN PERCENTAGE of GLRLM in VOI_edema in 256-leveled T2-weighted image.
188	T2_edema_GLRLMRln	RUN LENGTH NON-UNIFORMITY in VOI_edema in 256-leveled T2-weighted image.
189	T2_edema_GLRLMLrge	LOW GRAY LEVEL RUN EMPHASIS in VOI_edema in 256-leveled T2-weighted image.
190	T2_edema_GLRLMHrge	HIGH GRAY LEVEL RUN EMPHASIS in VOI_edema in 256-leveled T2-weighted image.
191	T2_edema_GLCMcontrast_1_SD	Standard deviation of contrast of GLCM in VOI_edema in 256-leveled T2-weighted image with offset set to 1.
192	T2_edema_GLCMcontrast_2_SD	Standard deviation of contrast of GLCM in VOI_edema in 256-leveled T2-weighted image with offset set to 2.
193	T2_edema_GLCMcontrast_3_SD	Standard deviation of contrast of GLCM in VOI_edema in 256-leveled T2-weighted image with offset set to 3.
194	T2_edema_GLCMenergy_1_SD	Standard deviation of energy of GLCM in VOI_edema in 256-leveled T2-weighted image with offset set to 1.
195	T2_edema_GLCMenergy_2_SD	Standard deviation of energy of GLCM in VOI_edema in 256-leveled T2-weighted image with offset set to 2.
196	T2_edema_GLCMenergy_3_SD	Standard deviation of energy of GLCM in VOI_edema in 256-leveled T2-weighted image with offset set to 3.
197	T2_edema_GLCMhomogeniety_1_SD	Standard deviation of homogeneity of GLCM in VOI_edema in 256-leveled T2-weighted image with offset set to 1.
198	T2_edema_GLCMhomogeniety_2_SD	Standard deviation of homogeneity of GLCM in VOI_edema in 256-leveled T2-weighted image with offset set to 2.
199	T2_edema_GLCMhomogeniety_3_SD	Standard deviation of homogeneity of GLCM in VOI_edema in 256-leveled T2-weighted image with offset set to 3.
200	T2_edema_GLRLMSre_SD	Standard deviation of Short run emphasis of GLRLM in VOI_edema in 256-leveled T2-weighted image.
201	T2_edema_GLRLMLre_SD	Standard deviation of Long run emphasis of GLRLM in VOI_edema in 256-leveled T2-weighted image.
202	T2_edema_GLRLMGln_SD	Standard deviation of GRAY LEVEL NON-UNIFORMITY of GLRLM in VOI_edema in 256-leveled T2-weighted image.
203	T2_edema_GLRLMRp_SD	Standard deviation of RUN PERCENTAGE of GLRLM in VOI_edema in 256-leveled T2-weighted image.
204	T2_edema_GLRLMRln_SD	Standard deviation of RUN LENGTH NON-UNIFORMITY in VOI_edema in 256-leveled T2-weighted image.

205	T2_edema_GLRLMLrge_SD	Standard deviation of LOW GRAY LEVEL RUN EMPHASIS in VOI_edema in 256-leveled T2-weighted image.
206	T2_edema_GLRLMHrge_SD	Standard deviation of HIGH GRAY LEVEL RUN EMPHASIS in VOI_edema in 256-leveled T2-weighted image.
207	T2_prewitt_rim_Mean	Mean of rim of VOI Prewitt filtered 1) 256-leveled T2-weighted image.
208	T2_prewitt_rim_SD	Standard deviation of Rim of VOI Prewitt filtered 1) 256-leveled T2-weighted image.
209	T2_prewitt_rim_Var	Variance of Rim of VOI Prewitt filtered 1) 256-leveled T2-weighted image.
210	T2_prewitt_rim_RMS	Root Mean Square of Rim of VOI Prewitt filtered 1) 256-leveled T2-weighted image.
211	T2_prewitt_rim_Max	Maximum of Rim of VOI Prewitt filtered 1) 256-leveled T2-weighted image.
212	T2_prewitt_rim_Min	Minimum of Rim of VOI Prewitt filtered 1) 256-leveled T2-weighted image.
213	T2_prewitt_rim_Median	Median of Rim of VOI Prewitt filtered 1) 256-leveled T2-weighted image.
214	T2_prewitt_rim_Mode	Mode of Rim of VOI Prewitt filtered 1) 256-leveled T2-weighted image.
215	T2_prewitt_rim_Entropy	Entropy of Rim of VOI Prewitt filtered 1) 256-leveled T2-weighted image.
216	T2_prewitt_rim_Kurtosis	Kurtosis of Rim of VOI Prewitt filtered 1) 256-leveled T2-weighted image.
217	T2_prewitt_rim_Skewness	Skewness of Rim of VOI Prewitt filtered 1) 256-leveled T2-weighted image.
218	T2_prewitt_rim_GLCMcontrast_1	Mean contrast of GLCM in Rim of VOI Prewitt filtered 1) 256-leveled T2-weighted image with offset set to 1.
219	T2_prewitt_rim_GLCMcontrast_2	Mean contrast of GLCM in Rim of VOI Prewitt filtered 1) 256-leveled T2-weighted image with offset set to 2.
220	T2_prewitt_rim_GLCMcontrast_3	Mean contrast of GLCM in Rim of VOI Prewitt filtered 1) 256-leveled T2-weighted image with offset set to 3.
221	T2_prewitt_rim_GLCMenergy_1	Mean energy of GLCM in Rim of VOI Prewitt filtered 1) 256-leveled T2-weighted image with offset set to 1.
222	T2_prewitt_rim_GLCMenergy_2	Mean energy of GLCM in Rim of VOI Prewitt filtered 1) 256-leveled T2-weighted image with offset set to 2.
223	T2_prewitt_rim_GLCMenergy_3	Mean energy of GLCM in Rim of VOI Prewitt filtered 1) 256-leveled T2-weighted image with offset set to 3.
224	T2_prewitt_rim_GLCMhomogeniety_1	Mean homogeneity of GLCM in Rim of VOI Prewitt filtered 1) 256-leveled T2-weighted image with offset set to 1.
225	T2_prewitt_rim_GLCMhomogeniety_2	Mean homogeneity of GLCM in Rim of VOI Prewitt filtered 1) 256-leveled T2-weighted image with offset set to 2.
226	T2_prewitt_rim_GLCMhomogeniety_3	Mean homogeneity of GLCM in Rim of VOI Prewitt filtered 1) 256-leveled T2-weighted image with offset set to 3.
227	T2_prewitt_rim_GLRLMSre	Short run emphasis of GLRLM in Rim of VOI Prewitt filtered 1) 256-leveled T2-weighted image.
228	T2_prewitt_rim_GLRLMLre	Long run emphasis of GLRLM in Rim of VOI Prewitt filtered 1) 256-leveled T2-weighted image.

229	T2_prewitt_rim_GLRLMGln	GRAY LEVEL NON-UNIFORMITY of GLRLM in Rim of VOI Prewitt filtered 1) 256-leveled T2-weighted image.
230	T2_prewitt_rim_GLRLMRp	RUN PERCENTAGE of GLRLM in Rim of VOI Prewitt filtered 1) 256-leveled T2-weighted image.
231	T2_prewitt_rim_GLRLMRln	RUN LENGTH NON-UNIFORMITY in Rim of VOI Prewitt filtered 1) 256-leveled T2-weighted image.
232	T2_prewitt_rim_GLRLMLrge	LOW GRAY LEVEL RUN EMPHASIS in Rim of VOI Prewitt filtered 1) 256-leveled T2-weighted image.
233	T2_prewitt_rim_GLRLMHrge	HIGH GRAY LEVEL RUN EMPHASIS in Rim of VOI Prewitt filtered 1) 256-leveled T2-weighted image.
234	T2_prewitt_rim_GLCMcontrast_1_SD	Standard deviation of contrast of GLCM in Rim of VOI Prewitt filtered 1) 256-leveled T2-weighted image with offset set to 1.
235	T2_prewitt_rim_GLCMcontrast_2_SD	Standard deviation of contrast of GLCM in Rim of VOI Prewitt filtered 1) 256-leveled T2-weighted image with offset set to 2.
236	T2_prewitt_rim_GLCMcontrast_3_SD	Standard deviation of contrast of GLCM in Rim of VOI Prewitt filtered 1) 256-leveled T2-weighted image with offset set to 3.
237	T2_prewitt_rim_GLCMenergy_1_SD	Standard deviation of energy of GLCM in Rim of VOI Prewitt filtered 1) 256-leveled T2-weighted image with offset set to 1.
238	T2_prewitt_rim_GLCMenergy_2_SD	Standard deviation of energy of GLCM in Rim of VOI Prewitt filtered 1) 256-leveled T2-weighted image with offset set to 2.
239	T2_prewitt_rim_GLCMenergy_3_SD	Standard deviation of energy of GLCM in Rim of VOI Prewitt filtered 1) 256-leveled T2-weighted image with offset set to 3.
240	T2_prewitt_rim_GLCMhomogeniety_1_SD	Standard deviation of homogeneity of GLCM in Rim of VOI Prewitt filtered 1) 256-leveled T2-weighted image with offset set to 1.
241	T2_prewitt_rim_GLCMhomogeniety_2_SD	Standard deviation of homogeneity of GLCM in Rim of VOI Prewitt filtered 1) 256-leveled T2-weighted image with offset set to 2.
242	T2_prewitt_rim_GLCMhomogeniety_3_SD	Standard deviation of homogeneity of GLCM in Rim of VOI Prewitt filtered 1) 256-leveled T2-weighted image with offset set to 3.
243	T2_prewitt_rim_GLRLMSre_SD	Standard deviation of Short run emphasis of GLRLM in Rim of VOI Prewitt filtered 1) 256-leveled T2-weighted image.
244	T2_prewitt_rim_GLRLMLre_SD	Standard deviation of Long run emphasis of GLRLM in Rim of VOI Prewitt filtered 1) 256-leveled T2-weighted image.
245	T2_prewitt_rim_GLRLMGln_SD	Standard deviation of GRAY LEVEL NON-UNIFORMITY of GLRLM in Rim of VOI Prewitt filtered 1) 256-leveled T2-weighted image.
246	T2_prewitt_rim_GLRLMRp_SD	Standard deviation of RUN PERCENTAGE of GLRLM in Rim of VOI Prewitt filtered 1) 256-leveled T2-weighted image.
247	T2_prewitt_rim_GLRLMRln_SD	Standard deviation of RUN LENGTH NON-UNIFORMITY in Rim of VOI Prewitt filtered 1) 256-leveled T2-weighted image.
248	T2_prewitt_rim_GLRLMLrge_SD	Standard deviation of LOW GRAY LEVEL RUN EMPHASIS in Rim of VOI Prewitt filtered 1) 256-leveled T2-weighted image.
249	T2_prewitt_rim_GLRLMHrge_SD	Standard deviation of HIGH GRAY LEVEL RUN EMPHASIS in Rim of VOI Prewitt filtered 1) 256-leveled T2-weighted image.

250	T2_prewitt_rim_GLCMcorrelation_1	Correlation of GLCM in Rim of VOI Prewitt filtered 1) 256-leveled T2-weighted image with offset set to 1.
251	T2_prewitt_rim_GLCMcorrelation_2	Correlation of GLCM in Rim of VOI Prewitt filtered 1) 256-leveled T2-weighted image with offset set to 2.
252	T2_prewitt_rim_GLCMcorrelation_3	Correlation of GLCM in Rim of VOI Prewitt filtered 1) 256-leveled T2-weighted image with offset set to 3.
253	T2_prewitt_rim_GLCMcorrelation_1_SD	Standard deviation of Correlation of GLCM in Rim of VOI Prewitt filtered 1) 256-leveled T2-weighted image with offset set to 1.
254	T2_prewitt_rim_GLCMcorrelation_2_SD	Standard deviation of Correlation of GLCM in Rim of VOI Prewitt filtered 1) 256-leveled T2-weighted image with offset set to 2.
255	T2_prewitt_rim_GLCMcorrelation_3_SD	Standard deviation of Correlation of GLCM in Rim of VOI Prewitt filtered 1) 256-leveled T2-weighted image with offset set to 3.
256	core_on_MNI_Total_Surface_Area	Total surface area (A) of core_on_MNI.
257	core_on_MNI_Total_Volume	Total volume (V) of core_on_MNI.
258	core_on_MNI_Compactness01	Value calculated by the following equation of core_on_MNI; $compactness1 = \frac{V}{\sqrt{\pi} * A^{\frac{2}{3}}}$
259	core_on_MNI_Compactness02	Value calculated by the following equation of core_on_MNI; $compactness2 = 36\pi \frac{A^2}{V^3}$
260	core_on_MNI_Spherical_Disporoportion	Value calculated by the following equation of core_on_MNI; $spherical\_disproportion = \frac{A}{4\pi * R^2} = \frac{A}{(6\sqrt{\pi} * V)^{\frac{2}{3}}}$
261	core_on_MNI_Sphericity	Value calculated by the following equation of core_on_MNI; $sphericity = \frac{(6\pi^2 V)^{\frac{2}{3}}}{A}$
262	core_on_MNI_Surface_to_Volume_ratio	Value calculated by the following equation of core_on_MNI; $surface\_volume\_ratio = \frac{A}{V}$
263	edema_on_MNI_Total_Surface_Area	Total surface area (A) of edema_on_MNI.
264	edema_on_MNI_Total_Volume	Total volume (V) of edema_on_MNI.
265	edema_on_MNI_Compactness01	Value calculated by the following equation of edema_on_MNI; $compactness1 = \frac{V}{\sqrt{\pi} * A^{\frac{2}{3}}}$
266	edema_on_MNI_Compactness02	Value calculated by the following equation of edema_on_MNI;

		$$compactness2 = 36\pi \frac{A^2}{V^3}$$
267	edema_on_MNI_Spherical_Disporoportion	Value calculated by the following equation of edema_on_MNI; $$spherical\_disproportion = \frac{A}{4\pi * R^2} = \frac{A}{(6\sqrt{\pi} * V)^{\frac{2}{3}}}$$
268	edema_on_MNI_Sphericity	Value calculated by the following equation of edema_on_MNI; $$sphericity = \frac{(6\pi^2 V)^{\frac{2}{3}}}{A}$$
269	edema_on_MNI_Surface_to_Volume_ratio	Value calculated by the following equation of edema_on_MNI; $$surface\_volume\_ratio = \frac{A}{V}$$
270	T1Gd_edema_GLCMcorrelation_1	Correlation of GLCM in VOI_edema in 256-leveled Gadolinium enhanced T1-weighted image with offset set to 1.
271	T1Gd_edema_GLCMcorrelation_2	Correlation of GLCM in VOI_edema in 256-leveled Gadolinium enhanced T1-weighted image with offset set to 2.
272	T1Gd_edema_GLCMcorrelation_3	Correlation of GLCM in VOI_edema in 256-leveled Gadolinium enhanced T1-weighted image with offset set to 3.
273	T1Gd_edema_GLCMcorrelation_1_SD	Standard deviation of Correlation of GLCM in VOI_edema in 256-leveled Gadolinium enhanced T1-weighted image with offset set to 1.
274	T1Gd_edema_GLCMcorrelation_2_SD	Standard deviation of Correlation of GLCM in VOI_edema in 256-leveled Gadolinium enhanced T1-weighted image with offset set to 2.
275	T1Gd_edema_GLCMcorrelation_3_SD	Standard deviation of Correlation of GLCM in VOI_edema in 256-leveled Gadolinium enhanced T1-weighted image with offset set to 3.
276	T1_core_Mean	Mean of VOI_core in 256-leveled T1-weighted image.
277	T1_core_SD	Standard deviation of VOI_core in 256-leveled T1-weighted image.
278	T1_core_Var	Variance of VOI_core in 256-leveled T1-weighted image.
279	T1_core_RMS	Root Mean Square of VOI_core in 256-leveled T1-weighted image.
280	T1_core_Max	Maximum of VOI_core in 256-leveled T1-weighted image.
281	T1_core_Min	Minimum of VOI_core in 256-leveled T1-weighted image.
282	T1_core_Median	Median of VOI_core in 256-leveled T1-weighted image.
283	T1_core_Mode	Mode of VOI_core in 256-leveled T1-weighted image.
284	T1_core_Entropy	Entropy of VOI_core in 256-leveled T1-weighted image.
285	T1_core_Kurtosis	Kurtosis of VOI_core in 256-leveled T1-weighted image.
286	T1_core_Skewness	Skewness of VOI_core in 256-leveled T1-weighted image.
287	T1_core_GLCMcontrast_1	Mean contrast of GLCM in VOI_core in 256-leveled T1-weighted image with offset set to 1.

288	T1_core_GLCMcontrast_2	Mean contrast of GLCM in VOI_core in 256-leveled T1-weighted image with offset set to 2.
289	T1_core_GLCMcontrast_3	Mean contrast of GLCM in VOI_core in 256-leveled T1-weighted image with offset set to 3.
290	T1_core_GLCMenergy_1	Mean energy of GLCM in VOI_core in 256-leveled T1-weighted image with offset set to 1.
291	T1_core_GLCMenergy_2	Mean energy of GLCM in VOI_core in 256-leveled T1-weighted image with offset set to 2.
292	T1_core_GLCMenergy_3	Mean energy of GLCM in VOI_core in 256-leveled T1-weighted image with offset set to 3.
293	T1_core_GLCMhomogeniety_1	Mean homogeneity of GLCM in VOI_core in 256-leveled T1-weighted image with offset set to 1.
294	T1_core_GLCMhomogeniety_2	Mean homogeneity of GLCM in VOI_core in 256-leveled T1-weighted image with offset set to 2.
295	T1_core_GLCMhomogeniety_3	Mean homogeneity of GLCM in VOI_core in 256-leveled T1-weighted image with offset set to 3.
296	T1_core_GLRLMSre	Short run emphasis of GLRLM in VOI_core in 256-leveled T1-weighted image.
297	T1_core_GLRLMLre	Long run emphasis of GLRLM in VOI_core in 256-leveled T1-weighted image.
298	T1_core_GLRLMGln	GRAY LEVEL NON-UNIFORMITY of GLRLM in VOI_core in 256-leveled T1-weighted image.
299	T1_core_GLRLMRp	RUN PERCENTAGE of GLRLM in VOI_core in 256-leveled T1-weighted image.
300	T1_core_GLRLMRln	RUN LENGTH NON-UNIFORMITY in VOI_core in 256-leveled T1-weighted image.
301	T1_core_GLRLMLrge	LOW GRAY LEVEL RUN EMPHASIS in VOI_core in 256-leveled T1-weighted image.
302	T1_core_GLRLMHrge	HIGH GRAY LEVEL RUN EMPHASIS in VOI_core in 256-leveled T1-weighted image.
303	T1_core_GLCMcontrast_1_SD	Standard deviation of contrast of GLCM in VOI_core in 256-leveled T1-weighted image with offset set to 1.
304	T1_core_GLCMcontrast_2_SD	Standard deviation of contrast of GLCM in VOI_core in 256-leveled T1-weighted image with offset set to 2.
305	T1_core_GLCMcontrast_3_SD	Standard deviation of contrast of GLCM in VOI_core in 256-leveled T1-weighted image with offset set to 3.
306	T1_core_GLCMenergy_1_SD	Standard deviation of energy of GLCM in VOI_core in 256-leveled T1-weighted image with offset set to 1.
307	T1_core_GLCMenergy_2_SD	Standard deviation of energy of GLCM in VOI_core in 256-leveled T1-weighted image with offset set to 2.
308	T1_core_GLCMenergy_3_SD	Standard deviation of energy of GLCM in VOI_core in 256-leveled T1-weighted image with offset set to 3.
309	T1_core_GLCMhomogeniety_1_SD	Standard deviation of homogeneity of GLCM in VOI_core in 256-leveled T1-weighted image with offset set to 1.
310	T1_core_GLCMhomogeniety_2_SD	Standard deviation of homogeneity of GLCM in VOI_core in 256-leveled T1-weighted image with offset set to 2.
311	T1_core_GLCMhomogeniety_3_SD	Standard deviation of homogeneity of GLCM in VOI_core in 256-leveled T1-weighted image with offset set to 3.
312	T1_core_GLRLMSre_SD	Standard deviation of Short run emphasis of GLRLM in VOI_core in 256-leveled T1-weighted image.

313	T1_core_GLRLMLre_SD	Standard deviation of Long run emphasis of GLRLM in VOI_core in 256-leveled T1-weighted image.
314	T1_core_GLRLMGln_SD	Standard deviation of GRAY LEVEL NON-UNIFORMITY of GLRLM in VOI_core in 256-leveled T1-weighted image.
315	T1_core_GLRLMRp_SD	Standard deviation of RUN PERCENTAGE of GLRLM in VOI_core in 256-leveled T1-weighted image.
316	T1_core_GLRLMRln_SD	Standard deviation of RUN LENGTH NON-UNIFORMITY in VOI_core in 256-leveled T1-weighted image.
317	T1_core_GLRLMLrge_SD	Standard deviation of LOW GRAY LEVEL RUN EMPHASIS in VOI_core in 256-leveled T1-weighted image.
318	T1_core_GLRLMHrge_SD	Standard deviation of HIGH GRAY LEVEL RUN EMPHASIS in VOI_core in 256-leveled T1-weighted image.
319	T1_edema_Mean	Mean of VOI_edema in 256-leveled T1-weighted image.
320	T1_edema_SD	Standard deviation of VOI_edema in 256-leveled T1-weighted image.
321	T1_edema_Var	Variance of VOI_edema in 256-leveled T1-weighted image.
322	T1_edema_RMS	Root Mean Square of VOI_edema in 256-leveled T1-weighted image.
323	T1_edema_Max	Maximum of VOI_edema in 256-leveled T1-weighted image.
324	T1_edema_Min	Minimum of VOI_edema in 256-leveled T1-weighted image.
325	T1_edema_Median	Median of VOI_edema in 256-leveled T1-weighted image.
326	T1_edema_Mode	Mode of VOI_edema in 256-leveled T1-weighted image.
327	T1_edema_Entropy	Entropy of VOI_edema in 256-leveled T1-weighted image.
328	T1_edema_Kurtosis	Kurtosis of VOI_edema in 256-leveled T1-weighted image.
329	T1_edema_Skewness	Skewness of VOI_edema in 256-leveled T1-weighted image.
330	T1_edema_GLCMcontrast_1	Mean contrast of GLCM in VOI_edema in 256-leveled T1-weighted image with offset set to 1.
331	T1_edema_GLCMcontrast_2	Mean contrast of GLCM in VOI_edema in 256-leveled T1-weighted image with offset set to 2.
332	T1_edema_GLCMcontrast_3	Mean contrast of GLCM in VOI_edema in 256-leveled T1-weighted image with offset set to 3.
333	T1_edema_GLCMenergy_1	Mean energy of GLCM in VOI_edema in 256-leveled T1-weighted image with offset set to 1.
334	T1_edema_GLCMenergy_2	Mean energy of GLCM in VOI_edema in 256-leveled T1-weighted image with offset set to 2.
335	T1_edema_GLCMenergy_3	Mean energy of GLCM in VOI_edema in 256-leveled T1-weighted image with offset set to 3.
336	T1_edema_GLCMhomogeniety_1	Mean homogeneity of GLCM in VOI_edema in 256-leveled T1-weighted image with offset set to 1.
337	T1_edema_GLCMhomogeniety_2	Mean homogeneity of GLCM in VOI_edema in 256-leveled T1-weighted image with offset set to 2.
338	T1_edema_GLCMhomogeniety_3	Mean homogeneity of GLCM in VOI_edema in 256-leveled T1-weighted image with offset set to 3.
339	T1_edema_GLRLMSre	Short run emphasis of GLRLM in VOI_edema in 256-leveled T1-weighted image.
340	T1_edema_GLRLMLre	Long run emphasis of GLRLM in VOI_edema in 256-leveled T1-weighted image.
341	T1_edema_GLRLMGln	GRAY LEVEL NON-UNIFORMITY of GLRLM in VOI_edema in 256-leveled T1-weighted image.

342	T1_edema_GLRLMRp	RUN PERCENTAGE of GLRLM in VOI_edema in 256-leveled T1-weighted image.
343	T1_edema_GLRLMRln	RUN LENGTH NON-UNIFORMITY in VOI_edema in 256-leveled T1-weighted image.
344	T1_edema_GLRLMLrge	LOW GRAY LEVEL RUN EMPHASIS in VOI_edema in 256-leveled T1-weighted image.
345	T1_edema_GLRLMHrge	HIGH GRAY LEVEL RUN EMPHASIS in VOI_edema in 256-leveled T1-weighted image.
346	T1_edema_GLCMcontrast_1_SD	Standard deviation of contrast of GLCM in VOI_edema in 256-leveled T1-weighted image with offset set to 1.
347	T1_edema_GLCMcontrast_2_SD	Standard deviation of contrast of GLCM in VOI_edema in 256-leveled T1-weighted image with offset set to 2.
348	T1_edema_GLCMcontrast_3_SD	Standard deviation of contrast of GLCM in VOI_edema in 256-leveled T1-weighted image with offset set to 3.
349	T1_edema_GLCMenergy_1_SD	Standard deviation of energy of GLCM in VOI_edema in 256-leveled T1-weighted image with offset set to 1.
350	T1_edema_GLCMenergy_2_SD	Standard deviation of energy of GLCM in VOI_edema in 256-leveled T1-weighted image with offset set to 2.
351	T1_edema_GLCMenergy_3_SD	Standard deviation of energy of GLCM in VOI_edema in 256-leveled T1-weighted image with offset set to 3.
352	T1_edema_GLCMhomogeniety_1_SD	Standard deviation of homogeneity of GLCM in VOI_edema in 256-leveled T1-weighted image with offset set to 1.
353	T1_edema_GLCMhomogeniety_2_SD	Standard deviation of homogeneity of GLCM in VOI_edema in 256-leveled T1-weighted image with offset set to 2.
354	T1_edema_GLCMhomogeniety_3_SD	Standard deviation of homogeneity of GLCM in VOI_edema in 256-leveled T1-weighted image with offset set to 3.
355	T1_edema_GLRLMSre_SD	Standard deviation of Short run emphasis of GLRLM in VOI_edema in 256-leveled T1-weighted image.
356	T1_edema_GLRLMLre_SD	Standard deviation of Long run emphasis of GLRLM in VOI_edema in 256-leveled T1-weighted image.
357	T1_edema_GLRLMGln_SD	Standard deviation of GRAY LEVEL NON-UNIFORMITY of GLRLM in VOI_edema in 256-leveled T1-weighted image.
358	T1_edema_GLRLMRp_SD	Standard deviation of RUN PERCENTAGE of GLRLM in VOI_edema in 256-leveled T1-weighted image.
359	T1_edema_GLRLMRln_SD	Standard deviation of RUN LENGTH NON-UNIFORMITY in VOI_edema in 256-leveled T1-weighted image.
360	T1_edema_GLRLMLrge_SD	Standard deviation of LOW GRAY LEVEL RUN EMPHASIS in VOI_edema in 256-leveled T1-weighted image.
361	T1_edema_GLRLMHrge_SD	Standard deviation of HIGH GRAY LEVEL RUN EMPHASIS in VOI_edema in 256-leveled T1-weighted image.
362	T2_edema_GLCMcorrelation_1	Correlation of GLCM in VOI_edema in 256-leveled T2-weighted image with offset set to 1.
363	T2_edema_GLCMcorrelation_2	Correlation of GLCM in VOI_edema in 256-leveled T2-weighted image with offset set to 2.
364	T2_edema_GLCMcorrelation_3	Correlation of GLCM in VOI_edema in 256-leveled T2-weighted image with offset set to 3.

365	T2_edema_GLCMcorrelation_1_SD	Standard deviation of Correlation of GLCM in VOI_edema in 256-leveled T2-weighted image with offset set to 1.
366	T2_edema_GLCMcorrelation_2_SD	Standard deviation of Correlation of GLCM in VOI_edema in 256-leveled T2-weighted image with offset set to 2.
367	T2_edema_GLCMcorrelation_3_SD	Standard deviation of Correlation of GLCM in VOI_edema in 256-leveled T2-weighted image with offset set to 3.
368	z_score_core_Mean	Mean of VOI_core in Gdzscore 2).
369	z_score_core_SD	Standard deviation of VOI_core in Gdzscore 2).
370	z_score_core_Var	Variance of VOI_core in Gdzscore 2).
371	z_score_core_RMS	Root Mean Square of VOI_core in Gdzscore 2).
372	z_score_core_Max	Maximum of VOI_core in Gdzscore 2).
373	z_score_core_Min	Minimum of VOI_core in Gdzscore 2).
374	z_score_core_Median	Median of VOI_core in Gdzscore 2).
375	z_score_core_Mode	Mode of VOI_core in Gdzscore 2).
376	z_score_core_Entropy	Entropy of VOI_core in Gdzscore 2).
377	z_score_core_Kurtosis	Kurtosis of VOI_core in Gdzscore 2).
378	z_score_core_Skewness	Skewness of VOI_core in Gdzscore 2).
379	z_score_core_GLCMcontrast_1	Mean contrast of GLCM in VOI_core in Gdzscore 2) with offset set to 1.
380	z_score_core_GLCMcontrast_2	Mean contrast of GLCM in VOI_core in Gdzscore 2) with offset set to 2.
381	z_score_core_GLCMcontrast_3	Mean contrast of GLCM in VOI_core in Gdzscore 2) with offset set to 3.
382	z_score_core_GLCMenergy_1	Mean energy of GLCM in VOI_core in Gdzscore 2) with offset set to 1.
383	z_score_core_GLCMenergy_2	Mean energy of GLCM in VOI_core in Gdzscore 2) with offset set to 2.
384	z_score_core_GLCMenergy_3	Mean energy of GLCM in VOI_core in Gdzscore 2) with offset set to 3.
385	z_score_core_GLCMhomogeniety_1	Mean homogeneity of GLCM in VOI_core in Gdzscore 2) with offset set to 1.
386	z_score_core_GLCMhomogeniety_2	Mean homogeneity of GLCM in VOI_core in Gdzscore 2) with offset set to 2.
387	z_score_core_GLCMhomogeniety_3	Mean homogeneity of GLCM in VOI_core in Gdzscore 2) with offset set to 3.
388	z_score_core_GLRLMSre	Short run emphasis of GLRLM in VOI_core in Gdzscore 2).
389	z_score_core_GLRLMLre	Long run emphasis of GLRLM in VOI_core in Gdzscore 2).
390	z_score_core_GLRLMGln	GRAY LEVEL NON-UNIFORMITY of GLRLM in VOI_core in Gdzscore 2).
391	z_score_core_GLRLMRp	RUN PERCENTAGE of GLRLM in VOI_core in Gdzscore 2).
392	z_score_core_GLRLMRln	RUN LENGTH NON-UNIFORMITY in VOI_core in Gdzscore 2).
393	z_score_core_GLRLMLrge	LOW GRAY LEVEL RUN EMPHASIS in VOI_core in Gdzscore 2).
394	z_score_core_GLRLMHrge	HIGH GRAY LEVEL RUN EMPHASIS in VOI_core in Gdzscore 2).
395	z_score_core_GLCMcontrast_1_SD	Standard deviation of contrast of GLCM in VOI_core in Gdzscore 2) with offset set to 1.
396	z_score_core_GLCMcontrast_2_SD	Standard deviation of contrast of GLCM in VOI_core in Gdzscore 2) with offset set to 2.

397	z_score_core_GLCMcontrast_3_SD	Standard deviation of contrast of GLCM in VOI_core in Gdzscore 2) with offset set to 3.
398	z_score_core_GLCMenergy_1_SD	Standard deviation of energy of GLCM in VOI_core in Gdzscore 2) with offset set to 1.
399	z_score_core_GLCMenergy_2_SD	Standard deviation of energy of GLCM in VOI_core in Gdzscore 2) with offset set to 2.
400	z_score_core_GLCMenergy_3_SD	Standard deviation of energy of GLCM in VOI_core in Gdzscore 2) with offset set to 3.
401	z_score_core_GLCMhomogeniety_1_SD	Standard deviation of homogeneity of GLCM in VOI_core in Gdzscore 2) with offset set to 1.
402	z_score_core_GLCMhomogeniety_2_SD	Standard deviation of homogeneity of GLCM in VOI_core in Gdzscore 2) with offset set to 2.
403	z_score_core_GLCMhomogeniety_3_SD	Standard deviation of homogeneity of GLCM in VOI_core in Gdzscore 2) with offset set to 3.
404	z_score_core_GLRLMSre_SD	Standard deviation of Short run emphasis of GLRLM in VOI_core in Gdzscore 2).
405	z_score_core_GLRLMLre_SD	Standard deviation of Long run emphasis of GLRLM in VOI_core in Gdzscore 2).
406	z_score_core_GLRLMGln_SD	Standard deviation of GRAY LEVEL NON-UNIFORMITY of GLRLM in VOI_core in Gdzscore 2).
407	z_score_core_GLRLMRp_SD	Standard deviation of RUN PERCENTAGE of GLRLM in VOI_core in Gdzscore 2).
408	z_score_core_GLRLMRln_SD	Standard deviation of RUN LENGTH NON-UNIFORMITY in VOI_core in Gdzscore 2).
409	z_score_core_GLRLMLrge_SD	Standard deviation of LOW GRAY LEVEL RUN EMPHASIS in VOI_core in Gdzscore 2).
410	z_score_core_GLRLMHrge_SD	Standard deviation of HIGH GRAY LEVEL RUN EMPHASIS in VOI_core in Gdzscore 2).
411	z_score_edema_Mean	Mean of VOI_edema in Gdzscore 2).
412	z_score_edema_SD	Standard deviation of VOI_edema in Gdzscore 2).
413	z_score_edema_Var	Variance of VOI_edema in Gdzscore 2).
414	z_score_edema_RMS	Root Mean Square of VOI_edema in Gdzscore 2).
415	z_score_edema_Max	Maximum of VOI_edema in Gdzscore 2).
416	z_score_edema_Min	Minimum of VOI_edema in Gdzscore 2).
417	z_score_edema_Median	Median of VOI_edema in Gdzscore 2).
418	z_score_edema_Mode	Mode of VOI_edema in Gdzscore 2).
419	z_score_edema_Entropy	Entropy of VOI_edema in Gdzscore 2).
420	z_score_edema_Kurtosis	Kurtosis of VOI_edema in Gdzscore 2).
421	z_score_edema_Skewness	Skewness of VOI_edema in Gdzscore 2).
422	z_score_edema_GLCMcontrast_1	Mean contrast of GLCM in VOI_edema in Gdzscore 2) with offset set to 1.
423	z_score_edema_GLCMcontrast_2	Mean contrast of GLCM in VOI_edema in Gdzscore 2) with offset set to 2.
424	z_score_edema_GLCMcontrast_3	Mean contrast of GLCM in VOI_edema in Gdzscore 2) with offset set to 3.
425	z_score_edema_GLCMenergy_1	Mean energy of GLCM in VOI_edema in Gdzscore 2) with offset set to 1.
426	z_score_edema_GLCMenergy_2	Mean energy of GLCM in VOI_edema in Gdzscore 2) with offset set to 2.
427	z_score_edema_GLCMenergy_3	Mean energy of GLCM in VOI_edema in Gdzscore 2) with offset set to 3.
428	z_score_edema_GLCMhomogeniety_1	Mean homogeneity of GLCM in VOI_edema in Gdzscore 2) with offset set to 1.
429	z_score_edema_GLCMhomogeniety_2	Mean homogeneity of GLCM in VOI_edema in Gdzscore 2) with offset set to 2.
430	z_score_edema_GLCMhomogeniety_3	Mean homogeneity of GLCM in VOI_edema in Gdzscore 2) with offset set to 3.

431	z_score_edema_GLRLMSre	Short run emphasis of GLRLM in VOI_edema in Gdzscore 2).
432	z_score_edema_GLRLMLre	Long run emphasis of GLRLM in VOI_edema in Gdzscore 2).
433	z_score_edema_GLRLMGln	GRAY LEVEL NON-UNIFORMITY of GLRLM in VOI_edema in Gdzscore 2).
434	z_score_edema_GLRLMRp	RUN PERCENTAGE of GLRLM in VOI_edema in Gdzscore 2).
435	z_score_edema_GLRLMRln	RUN LENGTH NON-UNIFORMITY in VOI_edema in Gdzscore 2).
436	z_score_edema_GLRLMLrge	LOW GRAY LEVEL RUN EMPHASIS in VOI_edema in Gdzscore 2).
437	z_score_edema_GLRLMHrge	HIGH GRAY LEVEL RUN EMPHASIS in VOI_edema in Gdzscore 2).
438	z_score_edema_GLCMcontrast_1_SD	Standard deviation of contrast of GLCM in VOI_edema in Gdzscore 2) with offset set to 1.
439	z_score_edema_GLCMcontrast_2_SD	Standard deviation of contrast of GLCM in VOI_edema in Gdzscore 2) with offset set to 2.
440	z_score_edema_GLCMcontrast_3_SD	Standard deviation of contrast of GLCM in VOI_edema in Gdzscore 2) with offset set to 3.
441	z_score_edema_GLCMenergy_1_SD	Standard deviation of energy of GLCM in VOI_edema in Gdzscore 2) with offset set to 1.
442	z_score_edema_GLCMenergy_2_SD	Standard deviation of energy of GLCM in VOI_edema in Gdzscore 2) with offset set to 2.
443	z_score_edema_GLCMenergy_3_SD	Standard deviation of energy of GLCM in VOI_edema in Gdzscore 2) with offset set to 3.
444	z_score_edema_GLCMhomogeniety_1_SD	Standard deviation of homogeneity of GLCM in VOI_edema in Gdzscore 2) with offset set to 1.
445	z_score_edema_GLCMhomogeniety_2_SD	Standard deviation of homogeneity of GLCM in VOI_edema in Gdzscore 2) with offset set to 2.
446	z_score_edema_GLCMhomogeniety_3_SD	Standard deviation of homogeneity of GLCM in VOI_edema in Gdzscore 2) with offset set to 3.
447	z_score_edema_GLRLMSre_SD	Standard deviation of Short run emphasis of GLRLM in VOI_edema in Gdzscore 2).
448	z_score_edema_GLRLMLre_SD	Standard deviation of Long run emphasis of GLRLM in VOI_edema in Gdzscore 2).
449	z_score_edema_GLRLMGln_SD	Standard deviation of GRAY LEVEL NON-UNIFORMITY of GLRLM in VOI_edema in Gdzscore 2).
450	z_score_edema_GLRLMRp_SD	Standard deviation of RUN PERCENTAGE of GLRLM in VOI_edema in Gdzscore 2).
451	z_score_edema_GLRLMRln_SD	Standard deviation of RUN LENGTH NON-UNIFORMITY in VOI_edema in Gdzscore 2).
452	z_score_edema_GLRLMLrge_SD	Standard deviation of LOW GRAY LEVEL RUN EMPHASIS in VOI_edema in Gdzscore 2).
453	z_score_edema_GLRLMHrge_SD	Standard deviation of HIGH GRAY LEVEL RUN EMPHASIS in VOI_edema in Gdzscore 2).
454	T1_edema_GLCMcorrelation_1	Correlation of GLCM in VOI_edema in 256-leveled T1-weighted image with offset set to 1.
455	T1_edema_GLCMcorrelation_2	Correlation of GLCM in VOI_edema in 256-leveled T1-weighted image with offset set to 2.
456	T1_edema_GLCMcorrelation_3	Correlation of GLCM in VOI_edema in 256-leveled T1-weighted image with offset set to 3.
457	T1_edema_GLCMcorrelation_1_SD	Standard deviation of Correlation of GLCM in VOI_edema in 256-leveled T1-weighted image with offset set to 1.
458	T1_edema_GLCMcorrelation_2_SD	Standard deviation of Correlation of GLCM in VOI_edema in 256-leveled T1-weighted image with offset set to 2.
459	T1_edema_GLCMcorrelation_3_SD	Standard deviation of Correlation of GLCM in VOI_edema in 256-leveled T1-weighted image with offset set to 3.

460	z_score_edema_GLCM correlation_1	Correlation of GLCM in VOI_edema in Gdzscore 2) with offset set to 1.
461	z_score_edema_GLCM correlation_2	Correlation of GLCM in VOI_edema in Gdzscore 2) with offset set to 2.
462	z_score_edema_GLCM correlation_3	Correlation of GLCM in VOI_edema in Gdzscore 2) with offset set to 3.
463	z_score_edema_GLCM correlation_1_SD	Standard deviation of Correlation of GLCM in VOI_edema in Gdzscore 2) with offset set to 1.
464	z_score_edema_GLCM correlation_2_SD	Standard deviation of Correlation of GLCM in VOI_edema in Gdzscore 2) with offset set to 2.
465	z_score_edema_GLCM correlation_3_SD	Standard deviation of Correlation of GLCM in VOI_edema in Gdzscore 2) with offset set to 3.
466	T1_core_GLCMcorrel ation_1	Correlation of GLCM in VOI_core in 256-leveled T1-weighted image with offset set to 1.
467	T1_core_GLCMcorrel ation_2	Correlation of GLCM in VOI_core in 256-leveled T1-weighted image with offset set to 2.
468	T1_core_GLCMcorrel ation_3	Correlation of GLCM in VOI_core in 256-leveled T1-weighted image with offset set to 3.
469	T1_core_GLCMcorrel ation_1_SD	Standard deviation of Correlation of GLCM in VOI_core in 256-leveled T1-weighted image with offset set to 1.
470	T1_core_GLCMcorrel ation_2_SD	Standard deviation of Correlation of GLCM in VOI_core in 256-leveled T1-weighted image with offset set to 2.
471	T1_core_GLCMcorrel ation_3_SD	Standard deviation of Correlation of GLCM in VOI_core in 256-leveled T1-weighted image with offset set to 3.
472	T2_core_GLCMcorrel ation_1	Correlation of GLCM in VOI_core in 256-leveled T2-weighted image with offset set to 1.
473	T2_core_GLCMcorrel ation_2	Correlation of GLCM in VOI_core in 256-leveled T2-weighted image with offset set to 2.
474	T2_core_GLCMcorrel ation_3	Correlation of GLCM in VOI_core in 256-leveled T2-weighted image with offset set to 3.
475	T2_core_GLCMcorrel ation_1_SD	Standard deviation of Correlation of GLCM in VOI_core in 256-leveled T2-weighted image with offset set to 1.
476	T2_core_GLCMcorrel ation_2_SD	Standard deviation of Correlation of GLCM in VOI_core in 256-leveled T2-weighted image with offset set to 2.
477	T2_core_GLCMcorrel ation_3_SD	Standard deviation of Correlation of GLCM in VOI_core in 256-leveled T2-weighted image with offset set to 3.
478	T1Gd_core_GLCMcorr elation_1	Correlation of GLCM in VOI_core in 256-leveled Gadolinium enhanced T1-weighted image with offset set to 1.
479	T1Gd_core_GLCMcorr elation_2	Correlation of GLCM in VOI_core in 256-leveled Gadolinium enhanced T1-weighted image with offset set to 2.
480	T1Gd_core_GLCMcorr elation_3	Correlation of GLCM in VOI_core in 256-leveled Gadolinium enhanced T1-weighted image with offset set to 3.
481	T1Gd_core_GLCMcorr elation_1_SD	Standard deviation of Correlation of GLCM in VOI_core in 256-leveled Gadolinium enhanced Gadolinium enhanced T1-weighted image with offset set to 1.
482	T1Gd_core_GLCMcorr elation_2_SD	Standard deviation of Correlation of GLCM in VOI_core in 256-leveled T2-weighted image with offset set to 2.
483	T1Gd_core_GLCMcorr elation_3_SD	Standard deviation of Correlation of GLCM in VOI_core in 256-leveled T2-weighted image with offset set to 3.

484	z_score_core_GLCMcorrelation_1	Correlation of GLCM in VOI_core in Gdzscore 2) with offset set to 1.
485	z_score_core_GLCMcorrelation_2	Correlation of GLCM in VOI_core in Gdzscore 2) with offset set to 2.
486	z_score_core_GLCMcorrelation_3	Correlation of GLCM in VOI_core in Gdzscore 2) with offset set to 3.
487	z_score_core_GLCMcorrelation_1_SD	Standard deviation of Correlation of GLCM in VOI_core in Gdzscore 2) with offset set to 1.
488	z_score_core_GLCMcorrelation_2_SD	Standard deviation of Correlation of GLCM in VOI_core in Gdzscore 2) with offset set to 2.
489	z_score_core_GLCMcorrelation_3_SD	Standard deviation of Correlation of GLCM in VOI_core in Gdzscore 2) with offset set to 3.

1) Prewitt filtering was performed by applying first order horizontal Gx and vertical Gy differentiation and by calculating the magnitude G, where Gx and Gy stands for the horizontal and vertical gradient of the image respectively and A for the original two-dimensional gray scale image

$$Gx = \begin{bmatrix} -1 & 0 & +1 \\ -1 & 0 & +1 \\ -1 & 0 & +1 \end{bmatrix} * A, \quad Gy = \begin{bmatrix} -1 & -1 & -1 \\ 0 & 0 & 0 \\ +1 & +1 & +1 \end{bmatrix} * A, \quad G = \sqrt{Gx^2 + Gy^2}$$

2) Gdzscore image was created by visualizing the magnitude of enhancement calculated from both 256-leveled non-enhanced and Gadolinium-enhanced T1-weighted images. 256-leveled Gadolinium-enhanced T1-weighted images was plotted as a function of 256-leveled non-enhanced T1-weighted images in whole brain. Linear regression fitting was applied to the data obtained, which can be expressed as follows;

$$(GdT1WI) = \alpha(T1WI) + \beta$$

where $(GdT1WI)$ and $(T1WI)$ are 256-leveled Gadolinium-enhanced and non-enhanced T1-weighted images. By solving α and β, one can now determine the linear correlation of 256-leveled Gadolinium-enhanced and non-enhanced T1-weighted images.
Next, the magnitude of deviation from the above solved linear regression line for any particular voxel (i) can be expressed as follows:

$$deviation_i = \frac{(GdT1WI)_i - \alpha(T1WI)_i - \beta}{\sqrt{\alpha^2 + 1}}$$

where $(GdT1WI)_i$ and $(T1WI)_i$ are the 256-leved values of voxel (i) in Gadolinium-enhanced and non-enhanced T1-weighted images.

Finally, the Gdzscore of each data point was defined as follows

$$Gdzscore_i = \frac{deviation_i - \mu}{\rho}$$

where μ and σ are the means and standard deviation of $deviation_i$ in the whole brain.
